# Time-resolved comparative genomics of ‘Candidatus Carsonella ruddii’ across psyllid lineages reveals a conserved core genome and contrasting secondary symbiont dynamics

**DOI:** 10.1099/mgen.0.001727

**Published:** 2026-06-12

**Authors:** Fengnian Wu, Kangyi Deng, Xiuxuan Lin, Xueying Wen, Yingchun Zhu, Shuyu Peng, Kainuo Cai, Suying Cai, Qinghan Wu, Xiaoting Zheng, Zhengchao Yu, Nan Mo, Hui Zhu, Yuzhong Zheng, Jianjian Huang, Yongqin Zheng, Eduardo G. P. Fox

**Affiliations:** 1 School of Life Sciences and Food Engineering, Hanshan Normal University, Chaozhou 521041, PR China; 2 Programa de Pós-Graduação em Ambiente e Sociedade (PPGAS), Universidade Estadual de Goiás (UEG), Quirinópolis, 75860-000, Goiás, Brazil

**Keywords:** ‘*Candidatus* Carsonella ruddii’, divergence dating, genome reduction, Psylloidea, secondary symbionts, symbionts

## Abstract

Psyllids harbour the obligate nutritional symbiont ‘*Candidatus* Carsonella ruddii’ (*Ca*Cr), yet complete *Ca*Cr genomes remain unevenly sampled across Psylloidea, limiting both comparative analysis and temporal inference. Here, seven complete *Ca*Cr genomes (165–174 kb) were assembled and annotated from psyllid hosts representing four families, including lineages for which genome-grade resources had previously been unavailable. We integrated whole-genome phylogenomics, pangenome analysis, fossil-calibrated relaxed-clock dating and quantitative PCR (based on 16S rRNA) screening of secondary symbionts. Across hosts, *Ca*Cr retained an extremely reduced, AT-rich (>82%) and gene-dense (>90% coding) genome architecture with a conserved core of 155 genes, while the accessory fraction was limited and lineage-specific. Most variable genes belonged to amino-acid metabolism or proteins of unknown function, suggesting differential erosion of peripheral functions around a stable translational and informational core. Phylogenomically, *Ca*Cr diversification broadly tracked deep host diversification, although the *Ca*Cr lineage from *Diaphorina citri* grouped with Triozidae-associated lineages rather than with other sampled Psyllidae. Using two host fossil-informed soft calibrations, we estimated that major *Ca*Cr divergences occurred mainly from the Paleogene to the Miocene, with crown diversification of *Cacopsylla*-associated *Ca*Cr at 15.95–18.99 Ma. In contrast to the stability of the primary symbiosis, secondary symbionts showed patchy host distributions: *Wolbachia-* and *Arsenophonus*-like lineages occurred in multiple hosts, ‘*Candidatus* Profftella armatura’ was restricted to *D. citri*, and no secondary symbiont was detected in *Cacopsylla chinensis*. These results provide a time-resolved comparative framework for *Ca*Cr evolution in psyllids and underscore the different evolutionary stability of primary and facultative associates.

**Impact Statement**

Psyllids depend on the obligate nutritional symbiont ‘*Candidatus* Carsonella ruddii’ (*Ca*Cr), but complete *Ca*Cr genomes remain sparse and phylogenetically uneven across Psylloidea. Here, we report seven complete circular *Ca*Cr genomes from hosts spanning four psyllid families, including lineages that previously lacked genome-grade resources. Comparative genomics shows that *Ca*Cr retains an extremely reduced, AT-rich, gene-dense core genome while still exhibiting lineage-specific differences in accessory genes and peripheral metabolic functions. Whole-genome phylogenomics, pangenome analysis and fossil-calibrated dating indicate that *Ca*Cr diversification broadly parallels deep host diversification, whereas secondary symbionts show a far more labile, mosaic distribution across hosts. These resources strengthen the comparative framework for psyllid symbiosis and clarify how minimal bacterial genomes persist and diverge during long-term obligate mutualism.

## Data Summary

The complete genome sequences of ‘*Candidatus* Carsonella ruddii’ recovered from *Diaphorina citri* (CP197248), *Bactericera cockerelli* (CP197249), *Cacopsylla citrisuga* (CP197250), *Cacopsylla chinensis* (CP197251), *Cornegenapsylla sinica* (CP197252), *Macrohomotoma gladiata* (CP197253) and *Blastopsylla occidentalis* (CP197254) have been deposited in GenBank under BioProject PRJNA1303861. Code S1 (genome-density calculations from PGAP annotations) and Code S2 (binary accessory-gene heatmap visualization) are provided with the online supplementary material. The authors confirm that all other supporting data, code and protocols have been provided within the article or in the supplementary material.

## Introduction

Heritable bacterial symbionts are central to the biology of psyllids, a diverse lineage of phloem-feeding Hemiptera whose nutritional ecology depends on microbial complementation [1, 2]. As in other sap-feeding insects, the contrast between ancient obligate nutritional symbionts and more labile facultative associates provides a useful lens for examining metabolic integration in insect–microbe mutualisms [1,3]. In psyllids, the obligate primary symbiont ‘*Candidatus* Carsonella ruddii’ (*Ca*Cr) resides in bacteriocytes and is widely regarded as the ancestral nutritional partner across Psylloidea [2,4, 5]. Like other long-term obligate symbionts, *Ca*Cr has undergone extreme genome reduction while retaining functions linked to essential amino-acid provisioning. For that reason, this system provides a valuable model for understanding how minimal bacterial genomes evolve under stable vertical transmission and prolonged metabolic integration [1,3, 6, 7].

Although psyllids include more than 4,000 described species and several major agricultural pests, genomic knowledge of their symbionts remains taxonomically uneven [8,9]. Survey work further suggests that psyllids often harbour additional secondary symbionts, commonly from *Enterobacteriaceae-* or *Wolbachia*-related lineages, but these associates vary markedly among hosts and appear to have been repeatedly gained, lost or replaced [10,11]. These secondary associates matter not only as components of the microbiome but also as potential contributors to host phenotype and ecological performance. In sap-feeding Hemiptera, such symbionts can affect defence, stress tolerance and other aspects of host biology, although their roles in psyllids are still unevenly characterized [12]. One well-studied psyllid example is *Diaphorina citri* (hereafter *D. citri*), in which the co-resident symbiont ‘*Candidatus* Profftella armatura’ produces the defensive polyketide diaphorin, illustrating that non-*Ca*Cr partners can make substantial contributions to psyllid biology [13,15]. *Ca*Cr, however, is consistently detected across psyllid lineages and, because it is inherited predominantly vertically, is expected to show broad host–symbiont codivergence over evolutionary time [2,4].

That expectation has been difficult to test at deeper phylogenetic scales because complete *Ca*Cr genomes are distributed unevenly across host lineages. At the time of dataset compilation, published genome-grade *Ca*Cr assemblies were available for only 11 psyllid host species and were concentrated in three families: Psyllidae, Triozidae and Aphalaridae (Table S1, available in the online Supplementary Material). Under the current family-level classification, Psylloidea comprises seven extant families (Aphalaridae, Calophyidae, Carsidaridae, Liviidae, Mastigimatidae, Psyllidae and Triozidae) [16]. Several major psyllid lineages, therefore, remained unsampled or poorly represented at the genome level. This includes families for which only short *Ca*Cr marker fragments had been reported; for example, Carsidaridae was represented only by short *Ca*Cr 16S rRNA fragments from six host species [11,17]. As a result, it has remained difficult to assess how strongly *Ca*Cr genome architecture is conserved across deep psyllid splits, whether lineage-specific gene losses parallel host diversification and how *Ca*Cr divergence times should be interpreted within a host-calibrated temporal framework.

These gaps matter because primary and secondary symbionts are expected to follow rather different evolutionary trajectories. Primary symbionts such as *Ca*Cr should mainly reflect long-term host association and genome erosion under vertical transmission [2,4], whereas facultative associates may better capture more recent ecological turnover, host switching or replacement [10,11]. A comparative framework spanning multiple psyllid families is, therefore, needed if the stable history of the primary symbiosis is to be separated from the more labile distribution of secondary partners.

Here, we assembled seven complete *Ca*Cr genomes from psyllid hosts representing four families, including the first complete *Ca*Cr genome from Carsidaridae and the first representative from the Aphalaridae subfamily Phacopteroninae. We combined comparative genomics, pangenome analysis, phylogenomics, fossil-calibrated relaxed-clock dating and 16S rRNA/quantitative PCR (qPCR) screening of secondary symbionts to address three related questions: (i) how structurally and functionally conserved *Ca*Cr genomes are across divergent hosts, (ii) to what extent *Ca*Cr phylogeny and divergence times are congruent with host diversification and (iii) how the evolutionary stability of the primary symbiosis compares with the host distribution of secondary symbionts.

## Methods

### Insect sampling and sequencing

**Fig. 1.**
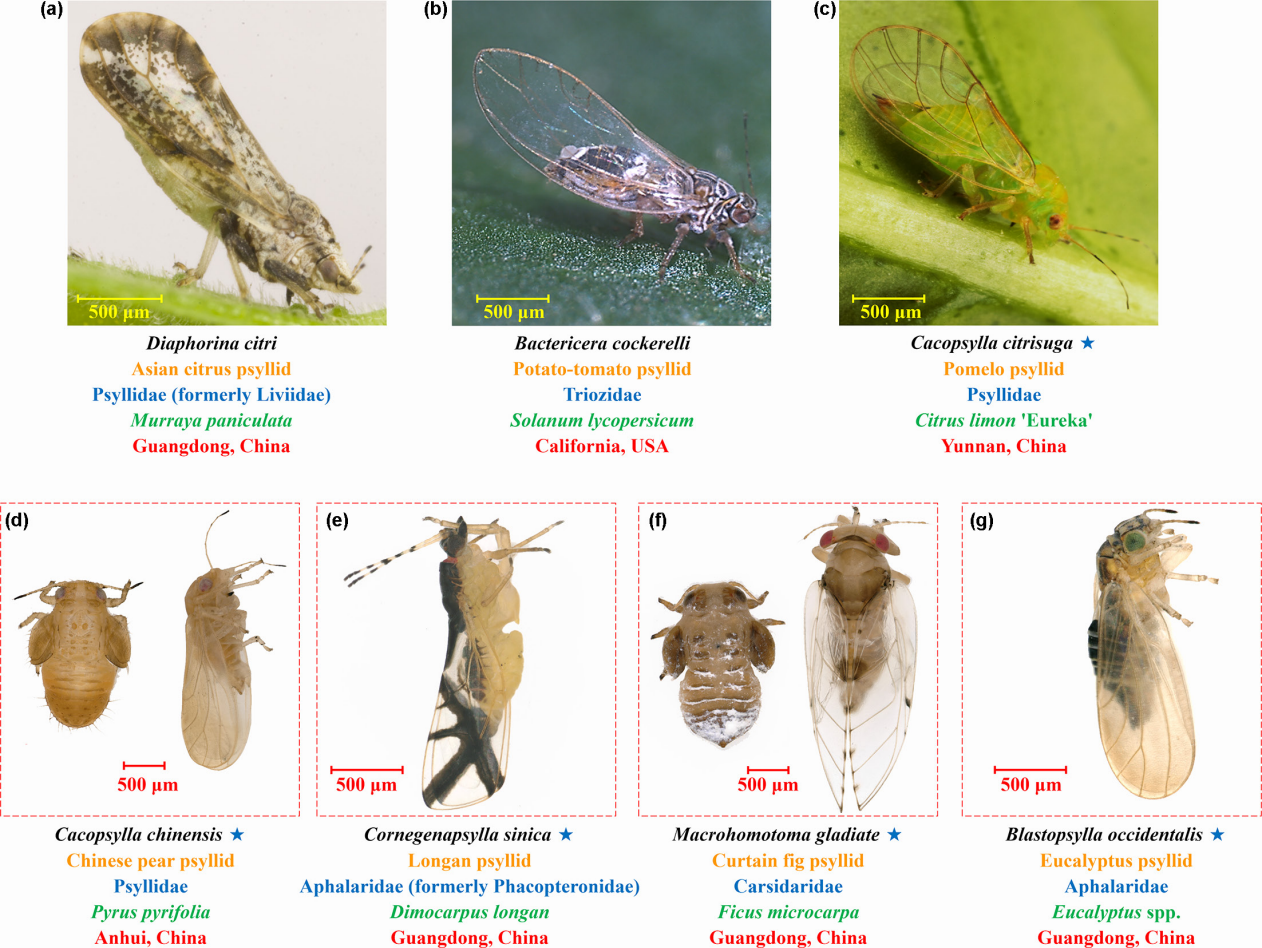
Representative specimens of seven psyllid species sampled for symbiont genome sequencing. While panels (a–c) and (e–g) show adults, panels (d) and (f) show a fifth-instar nymph (left) beside an adult (right). Blue asterisks indicate species for which no ‘*Candidatus* Carsonella ruddii’ genome assemblies were available at the time of this study (Table S1). Common names are shown in orange, family-level placements (following [16]) are in blue and host plants and sampling localities are in green and red, respectively.

Based on the available *Ca*Cr genome resources (Table S1) and the latest classification of Psylloidea [16], we selected seven psyllid species representing four families: Psyllidae, Triozidae, Aphalaridae and Carsidaridae (Fig. 1). Five of these species lacked published complete *Ca*Cr genomes at the time of sampling, whereas *D. citri* (Psyllidae; previously allocated in Liviidae) and the potato/tomato psyllid *Bactericera cockerelli* (hereafter *Bac. cockerelli*) (Triozidae) each already had published *Ca*Cr genomes [18,19]. Accordingly, the new dataset expands genomic sampling both taxonomically and phylogenetically, including the first complete *Ca*Cr genome from Carsidaridae and the first representative from the Aphalaridae subfamily Phacopteroninae.

Specimens and collection information are provided in Table S2. Specimens included (a) *D. citri* (Psyllidae), (b) *Bac. cockerelli* (Triozidae), (c) *Cacopsylla citrisuga* (hereafter *Cac. citrisuga*) (Psyllidae), (d) *Cacopsylla chinensis* (hereafter *Cac. chinensis*) (Psyllidae), (e) *Cornegenapsylla sinica* (hereafter *Cor. sinica*) (Aphalaridae), (f) *Macrohomotoma gladiata* (hereafter *M. gladiata*) (Carsidaridae) and (g) *Blastopsylla occidentalis* (hereafter *Bla. occidentalis*) (Aphalaridae). Species identification based on morphological characters and molecular barcoding followed Li [20] and Wu *et al.* [21].

Obtained specimens were preserved in 95% ethanol at −20 °C until use; total DNA (including nuclear, mitochondrial and symbiont DNA) was extracted from individual adults using the DNeasy Blood and Tissue Kit (QIAGEN) following the manufacturer’s instructions. DNA quality was assessed based on the following standard general parameters: integrity assessed by 1% agarose gel electrophoresis; purity as measured by NanoDrop absorbance range ratios A260/280=1.8–2.0, A260/230≥1.8; concentration ≥20 ng·μ^l−1^ by Qubit 4.0. A total of 60 µl of DNA within these parameters was obtained for each sample. Vouchers of specimens and DNA extracts are deposited at Hanshan Normal University. Next, genomic DNA was non-specifically amplified using the illustra GenomiPhi V2 DNA Amplification Kit (GE Healthcare Inc., Waukesha, WI, USA) to generate sufficient material for library preparation for next-generation sequencing on an Illumina HiSeq platform. The libraries were sequenced in March 2025 on an Illumina HiSeq platform to generate 150 bp paired-end reads, yielding >20 Gb raw data per sample.

### Read filtering and targeted assembly

The methods described in Wu *et al.* [19] enabled filtering out symbiont metagenomes from the host species-specific libraries. Towards this goal, the raw reads were quality-filtered and trimmed to form a pool of ‘clean’ reads, subjected to the software CLC Genomics Workbench v21 for generating draft *de novo* assemblies (under the parameters: word size=20; bubble size=50; minimum contig length=1,000 bp). After contig assembly, we compiled 11 publicly available *Ca*Cr genomes from GenBank (Table S1) and formatted them into a local Basic Local Alignment Search Tool (blast) database using blast+ (makeblastdb). The generated contigs were then subjected to blast+ (blastn) against this *Ca*Cr database locally to identify those candidate contigs of *Ca*Cr [22]. Following this filtering step, as *Ca*Cr is remarkably AT-rich and gets sequenced at lower coverage than the host mitochondrial genomes, we adopted a hybrid strategy in which two procedures were conducted in parallel: (i) mapping to the closest-matching *Ca*Cr metagenome reference by blastn (CLC mapping parameters: mismatch cost=2; insertion cost=3; deletion cost=3; length fraction=0.9; similarity fraction=0.9) to assemble the most conserved regions and (ii) *de novo* assembly to reconstruct regions departing from the references [6,7]. A consensus *Ca*Cr assembly for each sample was thus finally obtained by comparing the reference-guided and independent *de novo* assemblies and retaining the sequence version best supported by read coverage and more consistent paired-end links; pending ambiguous joins/conflicts were resolved by PCR and Sanger sequencing when necessary.

The draft *Ca*Cr assemblies were examined for circularity, and, as necessary, outward-facing primers were designed at some contig termini to generate PCR amplicons spanning a putative junction (the designed primer sequences and expected respective amplicon sizes are listed in Table S3). Sanger sequencing of the obtained amplicons provided bridging sequences resulting in circular chromosomes for all seven *Ca*Cr genomes. Following completion of final sequences, the Illumina reads were mapped to their respective *Ca*Cr assembly to compute per-base coverage and other mapping statistics for quality control.

### Gene annotation and genome-wide diversity analyses

The seven *Ca*Cr genomes were annotated using the National Center for Biotechnology Information (NCBI) Prokaryotic Genome Annotation Pipeline (PGAP) [23]. Basic genome statistics (genome size, gene counts and lengths and AT/GC content) were summarized with CLC Genomics Workbench v21. To quantify genome density, we used a Python script (Code S1) to parse PGAP feature coordinates and calculate gene overlap lengths and intergenic spacer lengths between adjacent annotated features. This script was used for descriptive genome summary calculations and related summary matrices.

Genome architecture was visualized with blast Ring Image Generator (BRIG) [24], using the *Ca*Cr genome assembled from *D. citri* (CP197248) as the reference; BRIG renders circular genome maps via CGView [25]. The resulting circular plots display annotated features together with GC content and GC skew. Pairwise average nucleotide identity (ANI) among the seven *Ca*Cr genomes was estimated with FastANI v1.33 (fragment length=1,000 bp) [26], and the resulting ANI matrix was visualized as a heatmap using custom Python scripts. In addition, whole-genome multiple sequence alignments (including conserved coding and intergenic regions) were generated in CLC Genomics Workbench using the ‘Very accurate’ setting (gap open cost=28.0; gap extension cost=1.0; end gap cost = ‘as any other’). Alignment gap patterns and conservation profiles across the seven genomes were inspected in CLC to locate those regions of elevated divergence.

For pangenome inference, the annotated GenBank flat files (.gbk) corresponding to accessions CP197248–CP197254 were converted into predicted ORF and protein FASTA files using genbank-to v0.42. Amino-acid sequences from all predicted coding genes were analysed with OrthoFinder v2.5.4 (default settings) to infer orthogroups and identify orthologous clusters [27]. Genes present in all seven genomes were treated as the core set, whereas all remaining genes were treated as accessory. Each accessory gene was then scored as present/absent in each genome based on blast+searches (*E*-value <1e-5) [22], generating a binary matrix that was visualized in R v4.4.1 with the pheatmap package using a custom R script (Code S2). Predicted protein sequences from both the core and accessory fractions were assigned provisional functional categories with eggNOG-mapper/Clusters of Orthologous Groups (COG) annotation [28].

### Phylogenetic and divergence-time analyses

A *Ca*Cr phylogenetic tree was constructed with the 18 sequenced lineages (i.e. 11 published genomes shown in Table S1+seven newly generated assemblies), rooted using two obligate P-symbionts as outgroups: (i) ‘*Candidatus* Nardonella dryophthoridicola’ strain NardRF (*Ca*Nd) and (ii) ‘*Candidatus* Portiera aleyrodidarum’ strain AF-CAI (*Ca*Pal). The first is a long-term, vertically transmitted P-symbiont of weevils recovered from the red palm weevil *Rhynchophorus ferrugineus* (Coleoptera: Curculionoidea: Curculionidae: Dryophthorinae; GenBank accession CP069383) [29], while the second is the primary nutritional symbiont in whiteflies, isolated from *Aleurodicus floccissimus* (Hemiptera: Sternorrhyncha: Aleyrodoidea: Aleyrodidae: Aleurodicinae; GenBank accession LN734649) [30].

Substitution-model selection was performed with ModelTest-NG v0.1.7 [31]. The concatenated alignment was analysed as a single partition, and the best-fitting nucleotide substitution model was selected for the full alignment. We inferred maximum-likelihood (ML) trees in PhyML 3.3.3.20220408 with 100 nonparametric bootstrap replicates [32]. Bayesian inference (BI) was carried out in MrBayes v3.2.7a, with two runs of four chains for 1,000,000 generations, sampling every 100 generations, where the first 25% were discarded as burn-in prior to computing posterior probabilities [33]. Convergence was assessed by the average standard deviation of split frequencies (<0.01) and potential scale reduction factors (~1.0).

Divergence times were estimated under a relaxed-clock framework using MCMCtree (PAML v4.10.9). The analysis employed concatenated *Ca*Cr nucleotide alignments as DNA data (seqtype=0) and an independent-rates model (clock=2; uncorrelated lognormal), with a birth-death-sampling tree prior BDparas=1 1 0 (lambda=1, mu=1, rho=0) [34,36]. We used the approximate-likelihood workflow of MCMCtree, first calculating gradients/Hessians (usedata=3) and then sampling the posterior (usedata=2). Calibration priors were limited to two fossil-informed soft bounds (Table S4). Because MCMCtree interprets calibrations on the time scale used in the tree file, the priors B(0.412, 0.478) and B(0.16, 0.19) correspond here to 41.2–47.8 Ma and 16–19 Ma, respectively, on a 100 Myr scale. These were implemented as soft-bounded priors rather than fixed node ages, allowing limited probability mass outside the specified interval to accommodate uncertainty in fossil assignment and stratigraphic age. The Aphalaridae calibration was informed by Eogyropsylla fossils associated with Lutetian deposits, whereas the *Cacopsylla* crown calibration followed the published Middle Miocene age of the Garang Formation and the fossil *Cacopsylla trigona*.

Two independent runs of 100 million steps were performed in MCMCtree, sampling every 10,000 steps, with the first 10% discarded as burn-in. Convergence and mixing were assessed in Tracer v1.7.2 using all key parameters with effective sample size (ESS) values>200 [37]. Log files from replicate runs were combined using LogCombiner (BEAST package), and FigTree was used to plot maximum-clade-credibility chronograms summarizing median node ages and their 95% highest posterior density intervals.

### Host phylogeny reconstruction and cophylogenetic analyses

To assess host–symbiont cophylogeny, we reconstructed the phylogeny of the psyllid hosts using mitochondrial cox1 sequences. Because *cox1* is the standardized DNA barcode marker for insects, it was the only genetic marker that could be obtained consistently for all sampled host taxa from GenBank or previous datasets. The dataset comprised *cox1* sequences from 18 psyllid species, including 11 host species associated with previously published *Ca*Cr genomes (Table S1) and seven host species analysed in the present study. To root the host tree, we included *cox1* sequences from *Rhynchophorus ferrugineus* and *Aleurodicus floccissimus* as outgroups. The *cox1* sequences for the seven newly sampled psyllids were obtained from Wu et al. [21]. Sequences were aligned in CLC Genomics Workbench. The host ML tree was inferred in PhyML using the GTR+G+I substitution model with 1000 bootstrap replicates. Bayesian inference (BI) was performed in MrBayes v3.2.7a with two runs of four chains for 1,500,000 generations, sampling every 100 generations. The resulting host topology was visualized in FigTree v1.4.4 [38].

To assess the overall congruence between host and *Ca*Cr phylogenies, we used ParaFit [39] with patristic distance matrices derived from the host *cox1* BI tree and the *Ca*Cr genome-based tree. We computed ParaFitGlobal with 999 permutations in the R package ape to evaluate the global *P*-value. We focused on the overall pattern of host–symbiont correspondence and, therefore, did not perform link-level tests for each host–symbiont association individually.

### Symbiont screening and prevalence assays (16S/qPCR)

*De novo*-assembled contigs from each host were screened using standalone blastn against a curated 16S rRNA reference database compiled from GenBank records retrieved on 15 December 2025 to catalogue the P-symbiont (*Ca*Cr) and any S-symbionts. Based on the taxa detected (Table S5), we designed SYBR Green qPCR primer sets producing 70–200 bp amplicons, together with conventional PCR primer sets producing 300–750 bp amplicons for confirmation. Primers for *Ca*Cr and *Wolbachia* were designed in conserved 16S rRNA regions to enable cross-host detection, whereas taxon-specific primer sets were used for other S-symbionts (Table S5). Primer specificity was assessed *in silico* by sequence similarity searches and empirically supported by single melt-curve peaks (qPCR) and/or single bands of the expected size (conventional PCR). For each psyllid species, 20 individuals were screened to estimate the infection status and prevalence of all detected symbionts. Prior to molecular assays, DNA quality was verified by requiring A260/A280 ratios of 1.7–2.0, A260/A230 ratios≥1.8 and DNA concentrations≥10 ng·µl⁻¹; samples failing these criteria were re-sampled and re-extracted until the standards were met. qPCR assays were performed using Bestar^®^ DBI SYBR Green chemistry (DBI Bioscience, Shanghai) following the manufacturer’s instructions and MIQE recommendations for reporting and quality control [40]. All qPCR reactions were run in technical triplicate, and each run included no-template controls (nuclease-free water) and an insect-DNA negative control. Samples yielding *Ct*≤28 with typical sigmoidal amplification curves were regarded as positive. Samples with undetermined *Ct* values or *Ct*>35 across all technical replicates were considered negative, whereas samples with 28<*Ct*≤35 were subjected to verification by conventional PCR using the corresponding primer sets listed in Table S5.

## Results

### Sequencing output, genome recovery and structural features of *Ca*Cr

Whole-genome sequencing was performed for seven psyllid species spanning four families; six hosts were collected in China and one (*Bac. cockerelli*) in the USA (Fig. 1, Table S2). Across libraries, sequencing yielded 7.02×10^7^ to 8.85×10^7^ raw paired-end reads per sample, corresponding to 23.6–29.8 GB of FASTQ data (Table S6). These datasets were used to filter symbiont-derived reads and recover complete circular *Ca*Cr chromosomes for all seven hosts (Table 1).

**Table 1.** Overview of assembled genomes of ‘*Candidatus* Carsonella ruddii’, recovered as primary symbionts of different species of psyllids (Hemiptera: Sternorrhyncha: Psylloidea) through whole-body Illumina sequencing followed by BLAST-targeted bioinformatics. A+T content can be calculated as 100 − (G+C) frequencies. PCG, protein-coding genes

Genome feature	Psyllid host						
	Psyllidae	Triozidae	Psyllidae	Psyllidae	Aphalaridae	Carsidaridae	Aphalaridae
	*D. citri*	*Bac. cockerelli*	*Cac. citrisuga*	*Cac. chinensis*	*Cor. sinica*	*M. gladiata*	*Bla. occidentalis*
NCBI submission no.	CP197248	CP197249	CP197250	CP197251	CP197252	CP197253	CP197254
Genome size (bp)	174,018	173,966	169,003	168,970	172,068	165,724	166,875
Total gene coverage length (bp)	160,507	169,668	164,949	156,790	155,059	162,207	163,362
Coding density (%)	92.24	97.53	97.6	92.79	90.11	97.88	97.89
Number of intergenic regions	96	61	70	101	64	69	61
Intergenic region length range (bp)	1–1,036	1–262	1–652	1–1,191	1–6,796	1–478	1–352
C+G content (%)	17.6	14.8	14.6	14.9	15.3	15.9	15.3
Number of tRNAs	27	26	25	27	25	25	26
tRNA length (bp)	71–89	71–88	71–90	71–91	71–87	71–85	71–85
Length of rRNAs (5S, 16S, 23S) (bp)	109–2,789	106–2,788	108–2,790	109–2,789	102–2,805	113–2,798	99–2,797
Number of PCGs	207	204	201	213	200	195	196
PCG length (bp)	114–3,876	114–3,873	114–3,885	96–3,882	114–3,891	114–3,849	72–3,861

The seven *Ca*Cr chromosomes are highly reduced (165,724–174,018 bp) and strongly AT-biassed (82.4–85.4% A+T, Table 1), with high coding density (90.11–97.89%). Each genome encodes 195–213 protein-coding genes as well as a conserved set of 25–27 tRNAs and 3 rRNA genes (5S, 16S and 23S) (Table 1). Genome organization was broadly conserved across different hosts (Fig. S1), and read mapping showed even coverage across the circular assemblies (mean depth 193×–72,903×, Fig. S1), leaving no extensive uncovered regions. Intergenic spacers are generally short, but their extent varies among lineages; notably, the maximum intergenic spacer length ranges from 262 bp in *Bac. cockerelli* to 6,796 bp in *Cor. sinica* (Table 1).

Our sampling added the first complete *Ca*Cr genome from Carsidaridae (*M. gladiata*) and the first representative from Phacopteroninae (*Cor. sinica*) (Tables 1 and S7). These newly sampled lineages were among the most divergent relative to previously published *Ca*Cr genomes.

### Comparative genomic alignment and nucleotide divergence of *Ca*Cr genomes

**Fig. 2.**
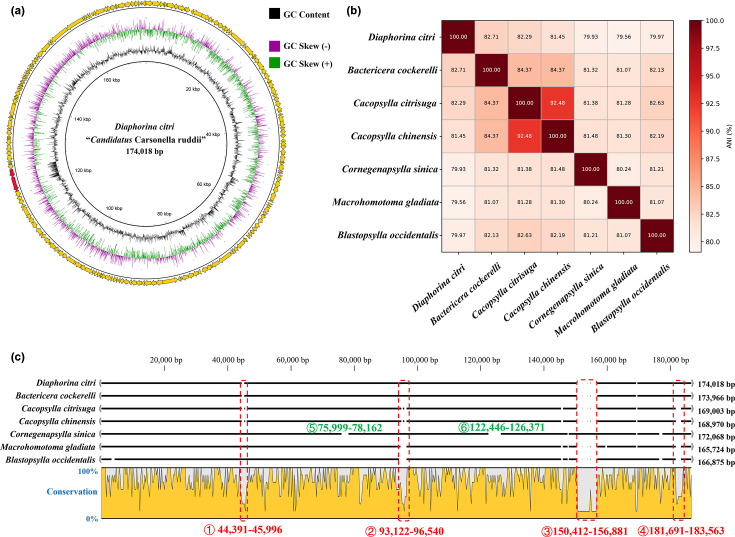
Comparative genome structures and nucleotide-level divergences across seven *Ca*Cr symbiont genomes. (**a**) Map of the *D. citri Ca*Cr genome (NCBI no. CP197248) set as reference, emphasizing GC content and skew in different colours. (**b**) Heatmap of pairwise ANI (in %) among cross-compared genomes. (**c**) Whole-genome alignment patterns where the most variable loci are emphasized within dotted red boxes, and green lines delineate genome-specific divergent loci (with coordinates of the aligned sections).

Whole-genome comparisons revealed a markedly conserved genomic framework among all assembled *Ca*Cr genomes. Using the *D. citri Ca*Cr genome as the reference (Fig. 2a), alignment of the multiple genomes was broadly collinear, with divergence concentrated in only a few loci (Fig. 2c). The GC-content across the reference genome remained even, showing only modestly limited deviations from a low baseline, whereas GC skew oscillated around zero and did not show long continuous regions of consistent sign (Fig. 2a).

Consistent with the returned alignment patterns, pairwise ANI values indicated heterogeneous divergence among different *Ca*Cr lineages (Fig. 2b), which, excluding self-to-self comparisons, ranged from 79.56 to 92.48%; two *Cacopsylla*-associated *Ca*Cr genomes returned the highest similarity observed (ANI=92.48%). Contrastingly, comparisons between sequences from more distantly-related hosts (notably the aphalarid- and carsidarid-associated lineages) yielded the lowest ANI values, reflecting deeper sequence divergence (Fig. 2b).

At a finer scale, the whole-genome alignments identified four hypervariable regions (Fig. 2c, red boxes) set against an otherwise highly conserved genomic background. In addition, two lineage-specific divergent segments (Fig. 2c, green boxes) were restricted to individual genomes, indicating that a small number of idiosyncratic loci have diverged within an otherwise conserved *Ca*Cr genome architecture.

### Pangenome structure and gene content variation among primary symbiont genomes

**Fig. 3.**
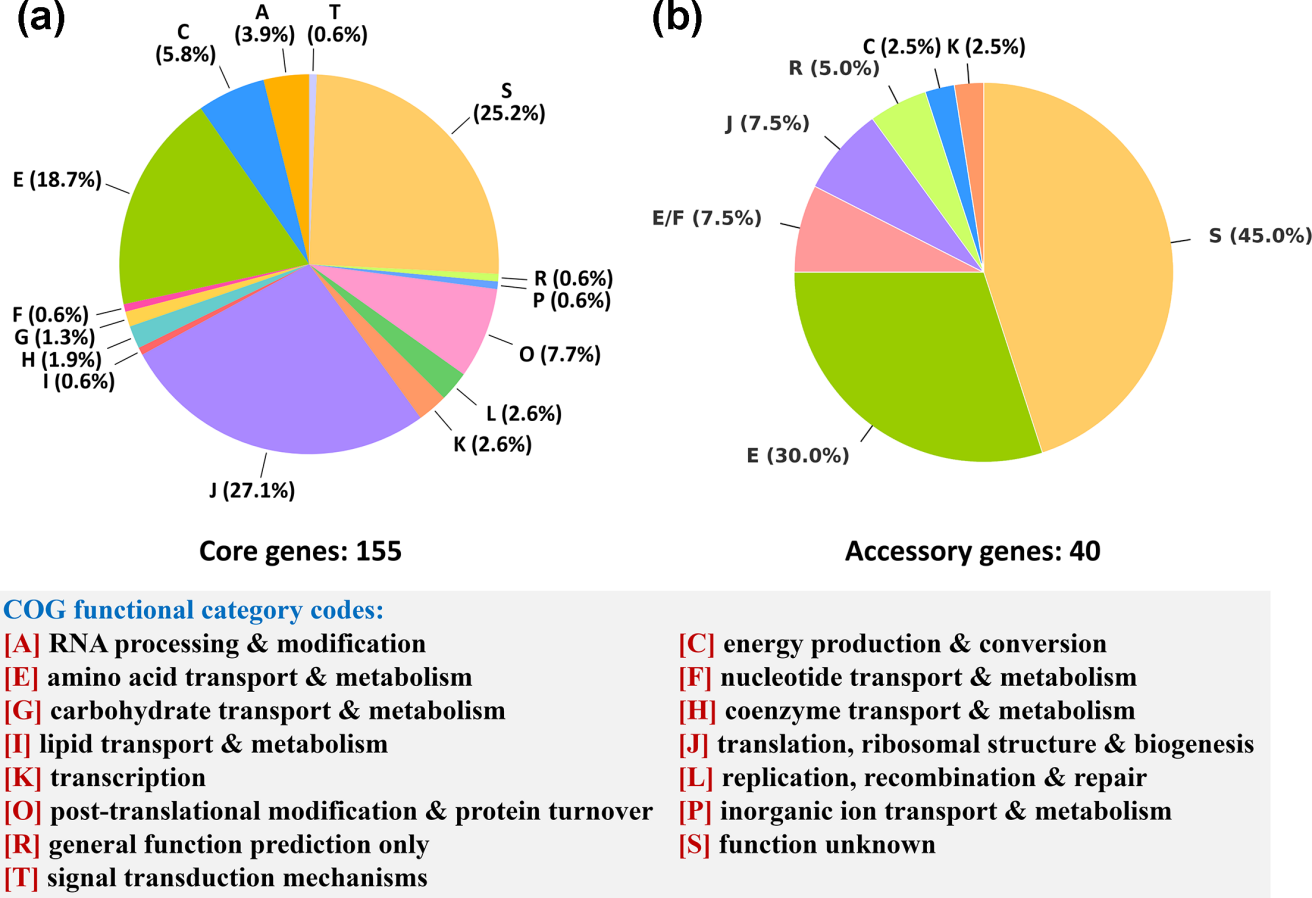
Functional classification (by COG colour codes, listed below the plots) of the core (**a**) and accessory genes (**b**) annotated from seven newly assembled *Ca*Cr genomes.

A total of 155 genes were shared by all 7 *Ca*Cr genomes and, therefore, constituted the core genome. Functionally, this core was dominated by genes involved in translation, ribosomal structure and biogenesis (42/155; 27.1%), followed by genes of unknown function (39/155; 25.2%) and amino-acid transport and metabolism (29/155; 18.7%) (Fig. 3a). By contrast, only 40 genes comprised the accessory fraction, and these were distributed patchily among lineages (Figs3b  4). Accessory genes were enriched in proteins of unknown function (45.0%) and amino-acid transport and metabolism (30.0%), indicating that the most evolutionarily labile part of the *Ca*Cr genome concerns peripheral nutritional functions rather than the informational core.

**Fig. 4.**
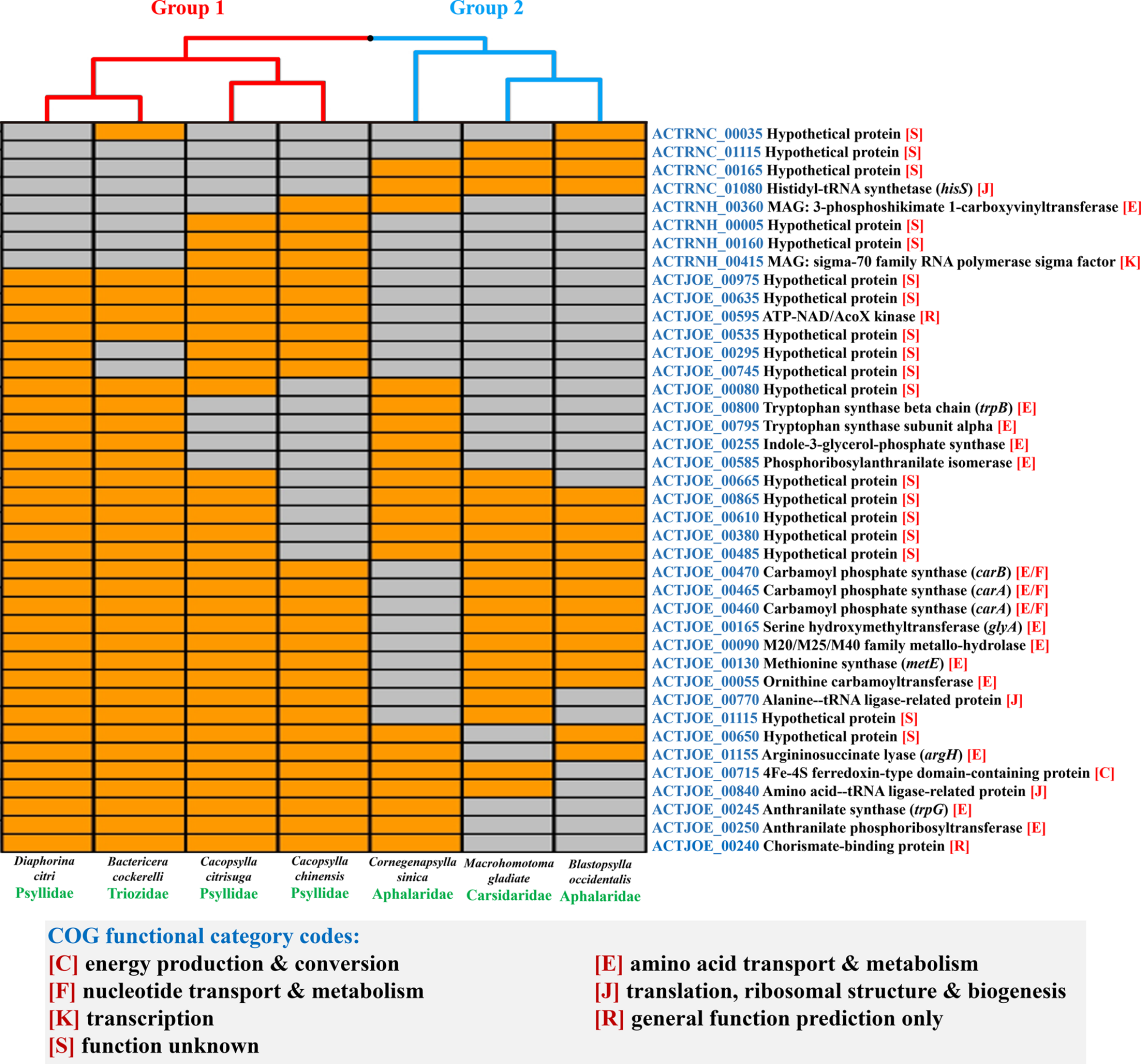
Binary matrix and hierarchical clustering of accessory-gene presence/absence across the seven *Ca*Cr genomes. Within the matrix, orange codes for the presence and grey for the absence of accessory genes (listed on the right-hand side) across seven newly assembled *Ca*Cr genomes from different psyllid hosts (host species and family names are given under each column). The matrix resulted in a dendrogram (drawn above the matrix) where the two major clusters (group 1, red; group 2, blue) are largely congruent with phylogenomic analyses depicted in Fig. 5. Gene names on the right side are followed by corresponding COG functional category codes in red, listed below the figure and as in Fig. 3.

**Fig. 5.**
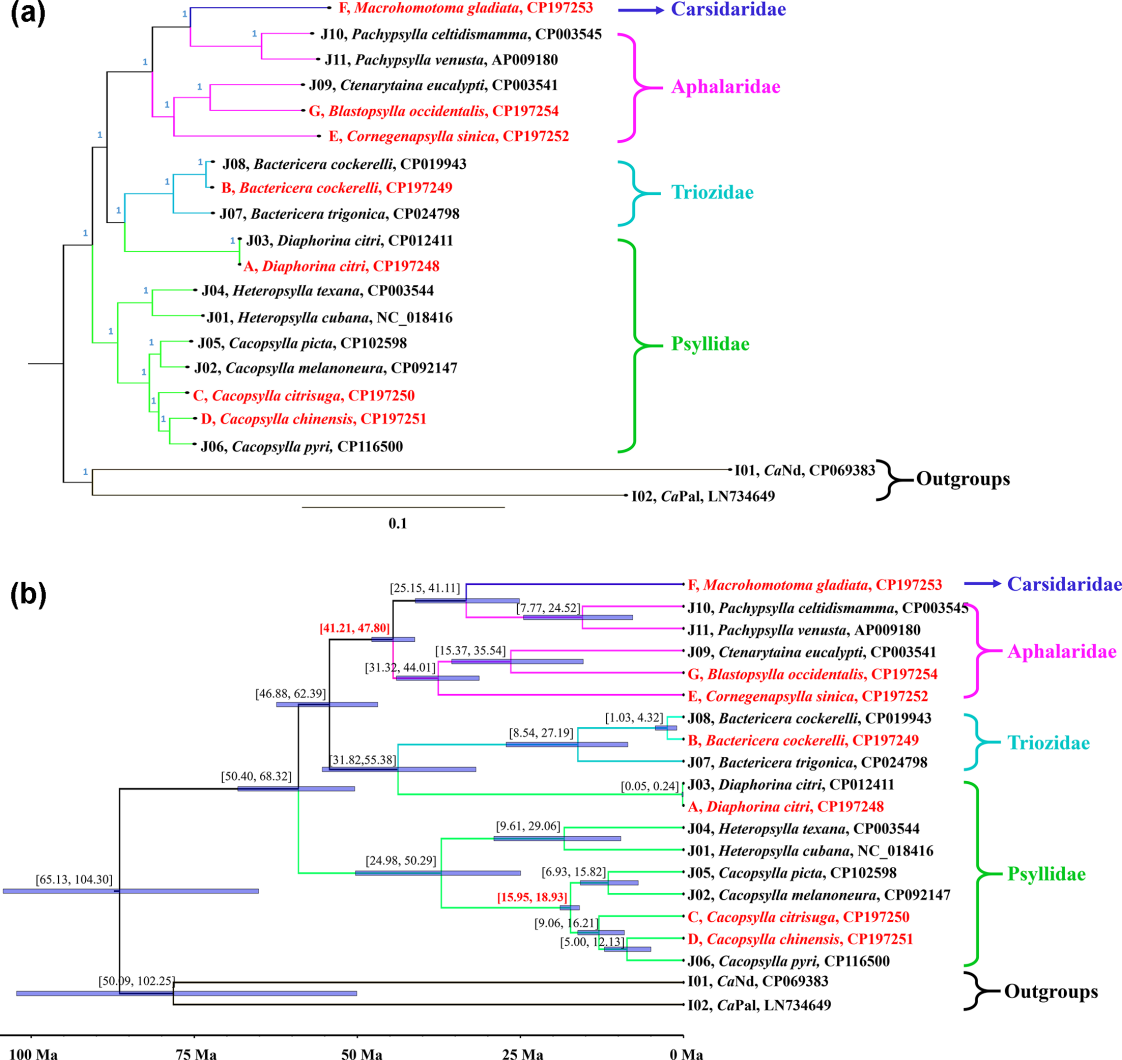
Phylogeny and divergence-time estimations for the primary symbiont *Ca*Cr recovered from psyllid hosts. (**a**) BI phylogeny reconstructed from *Ca*Cr genome alignments. Branches are colour-coded by host psyllid family (Carsidaridae, Aphalaridae, Triozidae and Psyllidae); the seven newly assembled genomes generated in this study are shown in red. Node labels indicate Bayesian posterior probabilities. The trees are rooted using ‘*Candidatus* Nardonella dryophthoridicola’ (*Ca*Nd) and ‘*Candidatus* Portiera aleyrodidarum’ (*Ca*Pal) as outgroups. (**b**) Time-calibrated chronogram generated under a relaxed-clock model in MCMCtree. Node estimated ages are given as medians (in Ma), with 95% highest posterior density (HPD) intervals displayed as horizontal node bars (i.e. credible-interval bars used in time trees). Fossil-calibrated nodes are marked with red (see Table S4 for calibration details).

The core genome retained multiple genes associated with amino-acid biosynthesis, including genes involved in histidine biosynthesis, the shikimate/aromatic-amino-acid backbone and the aspartate-family/branched-chain amino-acid network (Table S8), consistent with retention of a minimal nutritional backbone in *Ca*Cr. By contrast, several accessory genes mapped to terminal or peripheral biosynthetic steps and showed lineage-restricted distributions (Fig. 4). In particular, the tryptophan pathway genes *trpA*, *trpB*, indole-3-glycerol-phosphate synthase and phosphoribosylanthranilate isomerase were retained in the *D. citri*, *Bac. cockerelli* and *Cor. sinica* lineages but were absent from both sampled *Cacopsylla* lineages, *M. gladiata* and *Bla. occidentalis*. Likewise, *trpG* and anthranilate phosphoribosyltransferase were absent from *M. gladiata* and *Bla. occidentalis*, whereas *carA/carB*, ornithine carbamoyltransferase and *metE* were absent from *Cor. sinica*, and *argH* was absent from *M. gladiata* (Fig. 4). Together, these patterns indicate that differences among *Ca*Cr lineages are concentrated in peripheral metabolic steps rather than in the highly conserved informational core.

Hierarchical clustering of genomes using only accessory-gene presence/absence patterns divided the seven *Ca*Cr genomes into two main groups: (i) *D. citri*, *Bac. cockerelli* and the two *Cacopsylla*-associated lineages and (ii) *Cor. sinica*, *M. gladiata* and *Bla. occidentalis* (Fig. 4). This grouping is broadly concordant with the sequence-based phylogeny (Fig. 5), indicating that lineage-specific differential retention or loss of accessory genes carries a historical signal rather than representing extensive horizontal innovation.

### Phylogenetic relationships, divergence dating and host–symbiont correspondence

Phylogenetic analyses of *Ca*Cr yielded identical BI and ML topologies from the whole-genome dataset (Figs 5 and S2), with maximal or near-maximal internal support throughout. Aphalaridae-associated and Carsidaridae-associated lineages formed a clade in which the *Ca*Cr from *M. gladiata* was sister to sampled Aphalaridae lineages, including *Bla. occidentalis* and *Cor. sinica*. The *Ca*Cr lineages from *Bla. occidentalis* and *Cor. sinica* formed a sister pair. Within the remaining clade, however, the *Ca*Cr lineage from *D. citri* was recovered as sister to Triozidae-associated *Bactericera* lineages rather than to the sampled Psyllidae-associated *Cacopsylla* and *Heteropsylla* lineages.

Divergence-time estimation used two host fossil-informed soft calibrations: a Lutetian constraint for Aphalaridae and a Middle Miocene constraint for the crown of *Cacopsylla* (Fig. 5b, Table S4). Under this framework, the split between the sampled Psyllidae-associated *Ca*Cr lineages and the remaining *Ca*Cr lineages was estimated at 50.40–68.32 Ma. The divergence of the Aphalaridae+Carsidaridae clade from the Triozidae+*D. citri* clade was dated to 46.88–62.39 Ma, and the split of *D. citri Ca*Cr from other Triozidae-associated *Ca*Cr lineages was estimated at 31.82–55.38 Ma. Within the Aphalaridae+Carsidaridae clade, the *M. gladiata* lineage diverged from sampled Aphalaridae around 25.15–41.11 Ma, whereas the crown age of the sampled Aphalaridae-associated *Ca*Cr lineages was estimated at 31.32–44.01 Ma. Within Psyllidae, the divergence between *Heteropsylla* and *Cacopsylla* was estimated at 24.98–50.29 Ma, and crown diversification of sampled *Cacopsylla*-associated *Ca*Cr was estimated at 15.95–18.99 Ma.

**Fig. 6.**
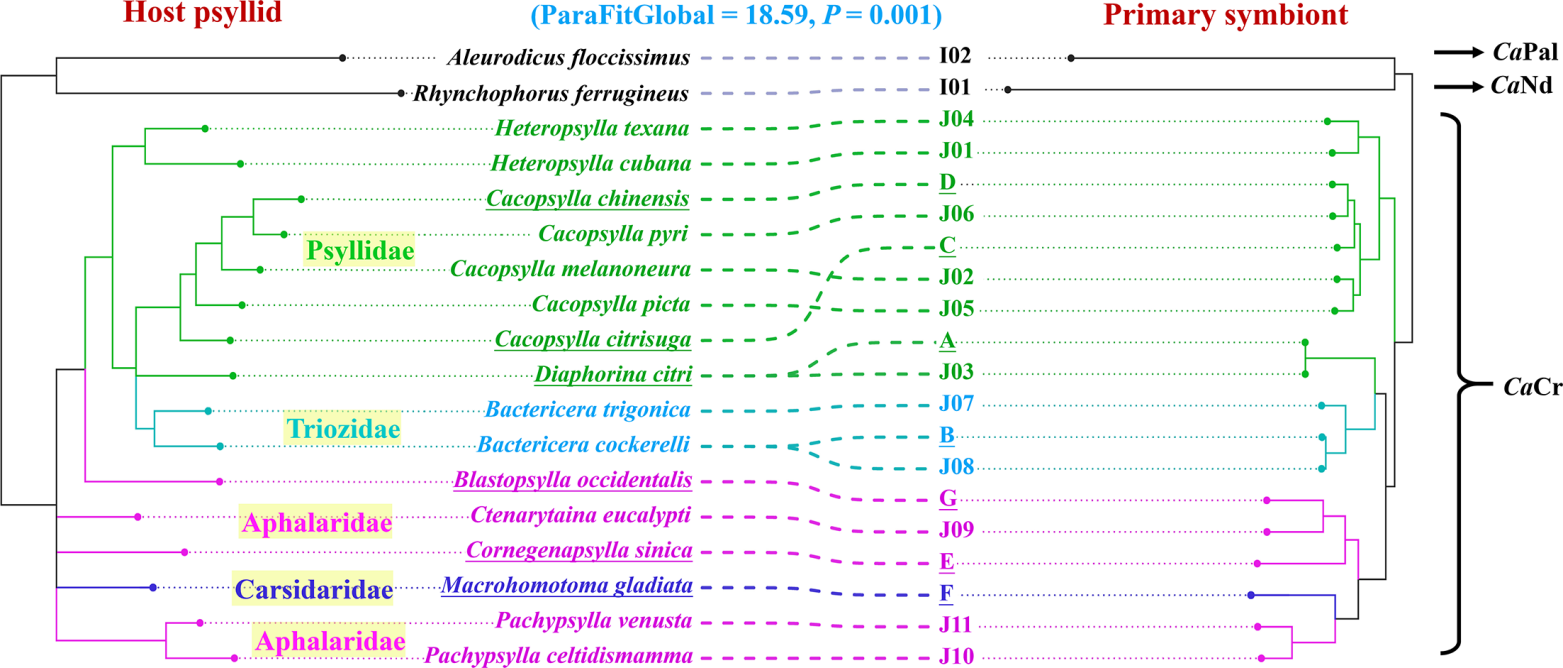
Topological congruence between the phylogenetic trees generated for psyllid hosts (left-hand side) and their primary symbiont *Ca*Cr (right-hand side) analysed with ParaFit. Both the host psyllids’ and primary symbiont’s phylogenies were generated with BI, but the first used mitochondrial *cox1* and the second used *Ca*Cr genomic data; they were compared using patristic distances. ParaFitGlobal and significance were evaluated by permutation testing (999 permutations). Coloured dashed links indicate observed host–symbiont associations, where the colours code for the host families Psyllidae, Triozidae, Aphalaridae and Carsidaridae. The *Ca*Cr tree was rooted using ‘*Candidatus* Nardonella dryophthoridicola’ (*Ca*Nd) and ‘*Candidatus* Portiera aleyrodidarum’ (*Ca*Pal) as outgroups, and the host tree was rooted using their respective insect hosts, *Rhynchophorus ferrugineus* and *Aleurodicus floccissimus*.

Comparison with the host phylogeny supported broad host–symbiont correspondence at deeper levels (Fig. 6). Major family-level groupings were largely mirrored between host and symbiont trees, and ParaFit supported significant non-random overall association between host and symbiont phylogenies (ParaFitGlobal=18.59, *P*=0.001; 999 permutations). At the same time, the *D. citri* association represented a localized incongruence; while the host belongs to Psyllidae, its *Ca*Cr lineage grouped with Triozidae-associated lineages. This pattern motivated a more cautious interpretation of codivergence in the ‘Discussion’ section and in the divergence-dating rationale.

### Distribution and prevalence of secondary symbionts across seven psyllid hosts

**Fig. 7.**
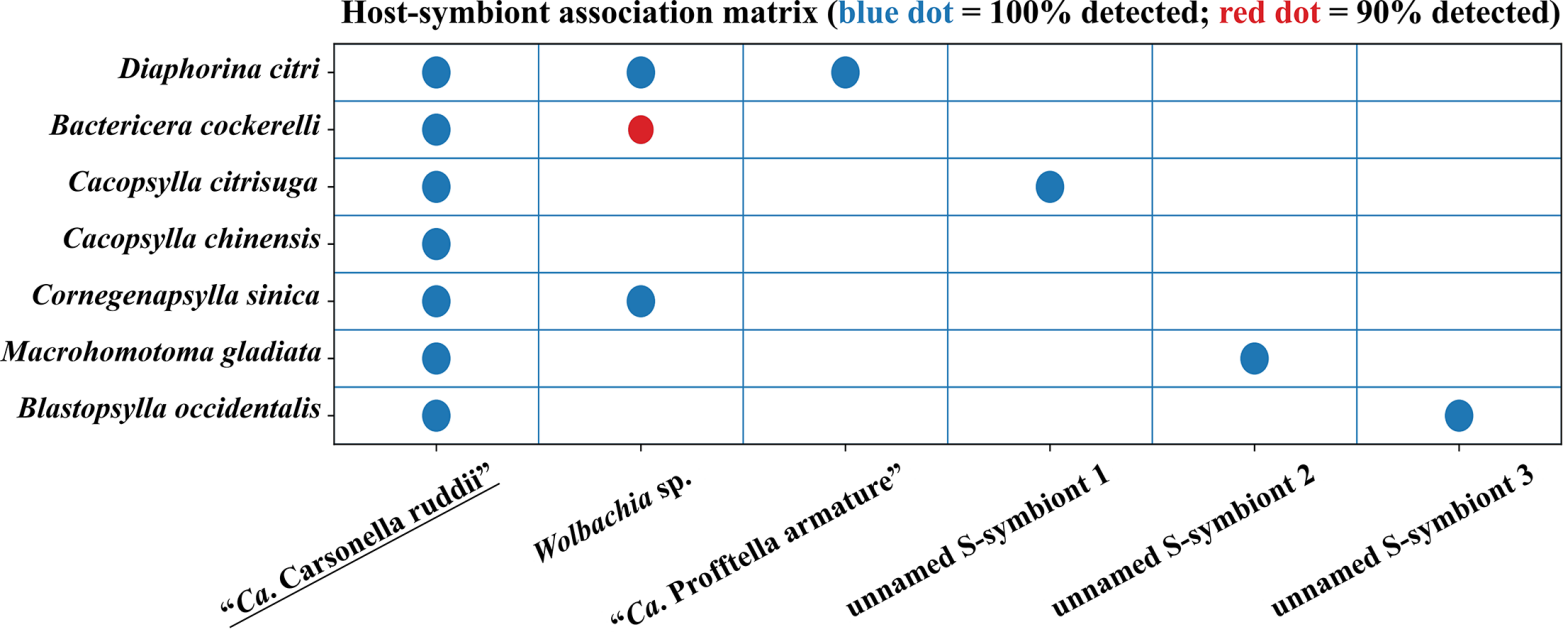
Detection prevalences of primary (first column, underlined) and secondary symbionts across seven inspected psyllid species. An association matrix summarizing symbiont prevalence per psyllid species. Samples were screened using quantitative PCR and conventional PCR with specific primers targeting the symbionts’ 16S rRNA gene (Table S5). For each psyllid species, 20 individuals were analysed. The coloured dots indicate presence, while their colour denotes detection frequency for each host–symbiont pair: blue, 100% detected (20/20); red, 90% detected (18/20).

Across all seven psyllid species, *Ca*Cr was detected in all (20/20) screened individuals per host species, consistent with its status as the universal primary symbiont in this dataset (Fig. 7, Table S9), whereas secondary symbionts showed host-specific and patchy distributions. *Wolbachia* was detected in *D. citri* (20/20), *Bac. cockerelli* (18/20) and *Cor. sinica* (20/20). blastn identification metrics for the corresponding amplicons are summarized in Table S9. ‘*Candidatus* Profftella armatura’ (16S–IGS–23S) was detected exclusively in *D. citri* and was present in all screened individuals (20/20), with full-length alignment to the reference sequence (Table S9).

*Cac. citrisuga*, *M. gladiata* and *Bla. occidentalis* each carried a single prevalent non-*Ca*Cr secondary symbiont, detected in all screened individuals (20/20, Fig. 7). The secondary symbiont from *Cac. citrisuga* had its best blastn hit to an Enterobacteriaceae bacterium annotated as ‘*Arsenophonus*-like’ (CP116499.1), whereas that from *M. gladiata* matched best to ‘*Candidatus* Arsenophonus nilaparvatae’ (CP158507.1) (Table S9). Because 16S identities for these lineages were only ~94–95%, below commonly used species-level thresholds, we conservatively refer to both as *Arsenophonus*-like lineages rather than assigning species names. By contrast, the symbiont recovered from *Bla. occidentalis* matched a previously deposited symbiont sequence from the same host at high identity (99.89%; AF263558.1), indicating the same or a very closely related lineage. No secondary symbiont was detected in *Cac. chinensis*. Overall, these results highlight the evolutionary stability of the primary symbiosis and the more dynamic distribution of facultative associates across psyllid hosts.

## Discussion

Complete genome resources for the obligate psyllid symbiont *Ca*Cr remain sparse and phylogenetically uneven. By expanding complete *Ca*Cr sampling across four psyllid families, we show that *Ca*Cr genome architecture is highly conserved across deep host splits, whereas accessory gene content and the occurrence of secondary symbionts are far more labile. Across divergent hosts, *Ca*Cr retained an extremely reduced, AT-rich, gene-dense genome, with only limited but informative variation in accessory genes and peripheral metabolic functions. This pattern is consistent with previous views of *Ca*Cr as a highly reduced obligate nutritional symbiont [6,7]. Taken together, the phylogenomic, temporal and screening data support broad – but not strict – host–symbiont codivergence, with a localized incongruence in *D. citri* and a much more dynamic pattern among secondary associates.

### Conservation of a reduced *Ca*Cr genome across divergent psyllid hosts

Across multiple psyllid families, the seven *Ca*Cr genomes were structurally conserved, preserving a small and highly compact chromosome with short intergenic regions, a strong AT bias and high coding density. The broad collinearity observed in whole-genome alignments, together with even read coverage and circularization supported by PCR and Sanger sequencing, argues against the possibility that the differences detected here are assembly artefacts. These features fit the longstanding view of *Ca*Cr as one of the most extremely reduced bacterial genomes known and, in some functional respects, an organelle-like nutritional partner [6,7, 41,43].

Even so, nucleotide divergence among lineages was substantial, with pairwise ANI values falling as low as 79.56% between the most distant sampled hosts. What emerges is a conserved genomic backbone punctuated by localized hypervariable regions, implying that *Ca*Cr diversification has proceeded mainly through sequence divergence and lineage-specific erosion around a stable architecture rather than through extensive genome rearrangement.

The addition of Carsidaridae and Phacopteroninae extends *Ca*Cr comparative genomics beyond the previously overrepresented Psyllidae/Triozidae framework. Because these chromosomes were circularized, showed homogeneous read coverage and were further validated by PCR/Sanger sequencing, their greater divergence from previously available reference genomes is most reasonably interpreted as genuine lineage-specific differentiation rather than technical error. Their inclusion also suggests that substantial *Ca*Cr diversity remains undocumented outside the currently best-studied, largely pest-associated lineages.

### Gene content differences suggest lineage-specific erosion of peripheral metabolic functions

The pangenome results support the same general interpretation at the gene-content level. Among the 7 *Ca*Cr genomes, 155 genes constituted a conserved core dominated by translation-related functions and other informational machinery, while still retaining a recognizable nutritional backbone that included genes associated with histidine biosynthesis, the shikimate pathway and the aspartate-family/branched-chain amino-acid network. This agrees with the long-recognized view of *Ca*Cr as a nutritional symbiont whose genome is extremely reduced but still preserves a subset of biosynthetic functions important to sap-feeding hosts [7,44]. The accessory genome, by comparison, was small and patchily distributed, and several lineage-variable genes were associated with amino acid metabolism rather than the informational core. This pattern suggests that the most evolutionarily labile portion of the *Ca*Cr genome lies in peripheral metabolic steps, not in translation, transcription or replication. That inference also fits broader models of genome reduction in obligate symbionts, where a minimal functional core is retained but peripheral biosynthetic steps are preferentially lost once they can be complemented by the host or by co-resident symbionts [7,44].

Several lineage-specific patterns in our dataset are consistent with this view. Most notably, a downstream tryptophan module (including *trpA*, *trpB*, indole-3-glycerol-phosphate synthase and phosphoribosylanthranilate isomerase) was retained in the *D. citri*, *Bac. cockerelli* and *Cor. sinica* lineages, but was absent from both sampled *Cacopsylla* lineages, *M. gladiata* and *Bla. occidentalis*. Additional lineage-restricted absences affected other amino-acid-related genes, again suggesting that the most labile component of the *Ca*Cr genome involves peripheral nutritional functions rather than random genome-wide decay. Previous work in psyllids has shown that missing *Ca*Cr functions can be complemented either by host-encoded genes, including horizontally transferred genes expressed in bacteriomes, or by co-resident symbionts with metabolically complementary pathways [10,45, 46]. In other *Cacopsylla* species, for example, incomplete *Ca*Cr tryptophan biosynthesis is complemented by the co-primary symbiont Psyllophila [10,45].

That said, our data do not directly demonstrate metabolic complementation. What they show is gene presence/absence and symbiont distribution, not the actual partitioning of biosynthetic function among partners. This distinction is particularly important for the two sampled *Cacopsylla* species, because *Cac. citrisuga* carried an *Arsenophonus*-like secondary symbiont, whereas no secondary symbiont was detected in *Cac. chinensis* during our screening. The lineage-specific losses described here should, therefore, be treated as candidate signatures of functional partitioning among *Ca*Cr, the host and additional microbial partners, rather than as direct evidence of which partner currently performs each missing step.

### Broad codivergence with localized incongruence in *Diaphorina*

Given that *Ca*Cr is transmitted predominantly vertically, broad host–symbiont codivergence is expected, and our results support that expectation at deeper phylogenetic levels. The significant ParaFitGlobal result and the broad agreement between host and symbiont trees at the family level indicate a non-random evolutionary association, even if topological identity is not recovered at every terminal lineage.

The clearest localized incongruence involves *D. citri*, whose *Ca*Cr lineage grouped with Triozidae-associated lineages rather than with the sampled Psyllidae-associated lineages. We do not take this discordance to invalidate the temporal framework, because neither of the two fossil calibrations was placed on the *D. citri* lineage itself; both were instead assigned to host-defined nodes that were stable in the analysed topology. A more cautious interpretation is, therefore, that *Ca*Cr diversification broadly follows host diversification at deeper phylogenetic scales, while some terminal relationships remain unresolved. Possible explanations include limited resolution of a host tree based on a single mitochondrial marker (*cox1*), compositional or rate biases or historical uncertainty in the placement of *Diaphorina* within psyllid classification [8,16, 21]. Additional host phylogenomic sampling will be needed to distinguish among these alternatives.

The placement of *Cor. sinica* within the sampled Aphalaridae-associated *Ca*Cr clade, by contrast, agrees with current host classification and indicates that the broader host–symbiont signal remains informative despite this localized discordance.

The fossil-informed dating analysis provides a provisional temporal framework for *Ca*Cr diversification, but it should be interpreted with the assumptions of the calibration strategy in mind. Because the calibrations derive from host fossils rather than *Ca*Cr itself, the dating analysis is conditional on broad codivergence at the calibrated nodes. Treating these calibrations as soft bounds rather than fixed ages is, therefore, important because it accommodates both phylogenetic and stratigraphic uncertainty while still allowing time-resolved comparison of deep *Ca*Cr divergences.

### Secondary symbionts show much more dynamic host distributions than *Ca*Cr

Compared with the stability of *Ca*Cr, secondary symbionts showed strikingly patchy distributions across the seven hosts. *Wolbachia* was found in three hosts from different families, ‘*Candidatus* Profftella armatura’ was confined to *D. citri*, *Arsenophonus*-like lineages occurred in two phylogenetically distant hosts and no secondary symbiont was detected in Cac. chinensis. This mosaic distribution is consistent with the more dynamic evolutionary behaviour expected of facultative or replacement-prone associates.

The incomplete *Wolbachia* prevalence in *Bac. cockerelli* (18/20 individuals) suggests infection polymorphism, perhaps reflecting imperfect maternal transmission, recent spread or local ecological heterogeneity. More broadly, the recurrence of *Wolbachia-* and *Arsenophonus*-related bacteria in distinct psyllid hosts is consistent with repeated gain, loss and probably host switching, in marked contrast to the long-term vertical persistence of *Ca*Cr [10,11].

For now, however, our screening data speak primarily to distribution rather than function. The consequences of these secondary symbionts for host nutrition, defence or pathogen transmission, therefore, remain hypotheses for future work, not direct conclusions of the present study.

The absence of a detectable secondary symbiont in *Cac. chinensis* is also notable. Whether this reflects a genuine lack of persistent secondary associates, low-titre lineages below the detection threshold or geographically structured variation remains unresolved and will require broader sampling together with deeper metagenomic sequencing.

### Implications and broader relevance

These findings provide a comparative evolutionary framework for psyllid–symbiont associations across multiple host lineages. With seven new complete *Ca*Cr genomes, the present study shows more clearly how an extremely reduced primary symbiont can remain structurally conserved across deeply divergent hosts, even while facultative associates display far more dynamic and host-specific distributions. This contrast is relevant not only to psyllid symbiosis but also to broader questions of genome reduction, metabolic integration and the evolutionary consequences of symbiont gain, loss and replacement.

### Limitations and future directions

Several limitations should, however, be kept in mind. First, host phylogeny was reconstructed from cox1 because it was the only marker available for all taxa, so deeper relationships inferred from a single mitochondrial gene should be treated cautiously. Second, divergence-time estimation relied on two host-derived fossil calibrations and is, therefore, contingent on broad codivergence at the calibrated nodes. Third, the present study did not directly test gene function, expression or metabolite exchange, and interpretations of lineage-specific gene losses, therefore, remain provisional. Future work will need broader host and symbiont sampling, together with host phylogenomics, bacteriome transcriptomics and metabolic reconstruction, to determine how *Ca*Cr gene loss is compensated in different psyllid lineages and how facultative associates contribute to host biology. Although symbiont-targeted manipulation may eventually prove relevant to pest management, the present study is better viewed as a comparative framework and a set of testable hypotheses for future evolutionary and functional research.

## Supplementary material

10.1099/mgen.0.001727 Supplementary Material 1.

## Abbreviations

ANI: average nucleotide identity
BI: Bayesian inference
blast: Basic Local Alignment Search Tool
BRIG: blast Ring Image Generator
CaCr: *Candidatus* Carsonella ruddii
COG: Clusters of Orthologous Groups
ML: maximum-likelihood
NCBI: National Center for Biotechnology Information
PGAP: Prokaryotic Genome Annotation Pipeline
qPCR: quantitative PCR
