# Supplementary material for: Time-resolved comparative genomics of ‘Candidatus Carsonella ruddii’ across psyllid lineages reveals a conserved core genome and contrasting secondary symbiont dynamics
Source: Microb Genom. 2026 Jun 12;12(6):001727. doi: 10.1099/mgen.0.001727 (PMC13378792; doi:10.1099/mgen.0.001727)
Supplement: Supplementary Material 1. [file mgen-12-01727-s001.pdf]

Table S1. Summary of published endosymbiont genomes and corresponding host mitochondrial *cox1* sequences used in this study.

**Endosymbiont species**	**Id**	**Host family**	**Host species**	**Genbank accession**	**Reference**	**Total assemblies**[a]	**Host *cox1* accession**
"*Candidatus* Carsonella ruddii"	J01	Psyllidae	*Heteropsylla cubana*	NC_018416.1	Sloan & Moran, 2012	1	PV137639
	J02	Psyllidae	*Cacopsylla melanoneura*	CP092147.1	Unpublished	8	PV137632
	J03	Psyllidae	*Diaphorina citri*	CP146469.1	Wu et al., 2025	33	PP048971
	J04	Psyllidae	*Heteropsylla texana*	CP003544.1	Sloan & Moran, 2012	1	MT021798
	J05	Psyllidae	*Cacopsylla picta*	CP102598.1	Unpublished	2	PV137634
	J06	Psyllidae	*Cacopsylla pyri*	CP116500.1	Unpublished	1	PV137636
	J07	Triozidae	*Bactericera trigonica*	CP024798.1	Unpublished	1	OR027186
	J08	Triozidae	*Bactericera cockerelli*	CP019943.1	Riley et al., 2017	2	NC_030055
	J09	Aphalaridae	*Ctenarytaina eucalypti*	CP003541.1	Sloan & Moran, 2012	1	PX507507
	J10	Aphalaridae	*Pachypsylla* sp. 'celtidis'	CP003545.1	Sloan & Moran, 2012	1	MG988803
	J11	Aphalaridae	*Pachypsylla venusta*	AP009180.1	Nakabachi et al., 2006	1	AY278317
"*Ca.* Profftella armatura"	K1	Psyllidae	*Diaphorina citri*	CP012591.1	Wu et al., 2015	37	—
Wolbachia sp.	L1	Psyllidae	*Diaphorina citri*	CP051608.1	Petrone et al., 2022	7	—
Enterobacteriaceae bacterium	M1	Psyllidae	*Cacopsylla pyri*	CP116499.1	Dittmer et al., 2023	1	—

[a] Number of complete genomes available in GenBank as of January 2026.

Table S2. Representative species sequenced in this study and collection information.

Sample ID	Species	Family	Isolate code	Collection locality	Coordinates (latitude, longitude)	Host plant	Year
A	*Diaphorina citri*	Psyllidae[b]	DC-GDSTJP	Jinping District, Shantou, Guangdong, China	23.37 °N, 116.70 °E	Murraya paniculata	2024
B	*Bactericera cockerelli*	Triozidae	BC-CARS	Riverside, California, USA	33.97 °N, 117.33 °W	Solanum lycopersicum	2015
C	*Cacopsylla citrisuga*	Psyllidae	CCI-YNRL	Ruili, Yunnan, China	25.14 °N, 99.18 °E	Citrus limon 'Eureka'	2016
D	*Cacopsylla chinensis*	Psyllidae	CCH-AH	Anhui, China	34.44 °N, 116.37 °E	Pyrus pyrifolia	2017
E	*Cornegenapsylla sinica*	Aphalaridae[a]	CS-GDJY	Jieyang, Guangdong, China	23.51 °N, 116.50 °E	Dimocarpus longan	2022
F	*Macrohomotoma gladiata*	Carsidaridae	MG-GDGZ	Guangzhou, Guangdong, China	23.15 °N, 113.36 °E	Ficus microcarpa	2018
G	*Blastopsylla occidentalis*	Aphalaridae	BO-GDGZ	Guangzhou, Guangdong, China	23.16 °N, 113.36 °E	Eucalyptus spp.	2018

[a] Family assignment follows Burckhardt et al. (2021), unless otherwise indicated; *Cornegenapsylla sinica* is treated as Aphalaridae (Phacopteroninae), rather than Phacopteronidae.

[b] The placement of Diaphorina has been historically unstable; here it is treated as Psyllidae.

Table S3. Details of primers for detecting circularization of primary symbiont "*Candidatus* Carsonella ruddii" genomes in psyllids in this study.

**Psyllids**	**Region**	**Forward primer (5'-3')**	**Reverse primer (5'-3')**	**PCR product size (bp)**
Diaphorina citri	173,746-332	TAAAATTCCCCTCCCC	CTCTCTAAAAATTCCCC	605
Bactericera cockerelli	171,620-318	TCAGGTGCTGCAACTTATAATT	TCCTGGTTTTGCTAGTCTTGC	2,693
Cacopsylla citrisuga	167,977-1,370	AGTTACTCTTTGCCCATCTTGT	AGCATGACCAGCACCTATTA	2,391
Cacopsylla chinensis	166,423-1,369	TGTGGATCAGGAATAATTAGTGC	GCATGACCAGCACCAATTAT	3,916
Cornegenapsylla sinica	14,688-667	TGTAGCGATCTCCAACTGCC	ACCTCCTAAAGCTCCAATGC	815
Macrohomotoma gladiata	164,669-743	TGAATCCAAAACCTTTCGTATCA	TTTGTTGTTCCAGGAATAGAAGT	1,799
Blastopsylla occidentalis	165,882-1,368	TGCAGTCATAGTTGTTATAGCAA	CAGAATGCCCACCTCCTATTATT	2,362

Table S4. Fossil calibrations used in MCMCtree.

**Calibration ID**	**Calibrated node (host-defined)**	**Fossil taxon (specimen/context)**	**Deposit & locality**	**Calibration prior (soft)**	**References**
CAL-01 (Aphalaridae crown)	Crown Aphalaridae — Carsonella lineages associated with Aphalaridae sensu lato (hosts include *Pachypsylla*, *Ctenarytaina*, *Blastopsylla* in Fig. 5B)	*Eogyropsylla* spp., Eocene Aphalarinae in Baltic amber; plus *Eogyropsylla paveloctogenarius* (compression fossil) from the Kishenehn Formation (USNM 553523)	Baltic amber (Eocene; Lutetian–Priabonian); Kishenehn Fm., Coal Creek Member, Montana, USA (Lutetian)	B(0.412, 0.478) — soft bounds spanning the Lutetian age window (middle Eocene)	Drohojowska, 2011; Ouvrard et al., 2013
CAL-02 (*Cacopsylla* crown)	Crown *Cacopsylla* — Carsonella lineages hosted by *Cacopsylla* (Fig. 5B)	*Cacopsylla trigona* sp. nov. (holotype) from the Garang Formation, Zeku County, E. Qinghai, China	Garang Formation (lacustrine–fluvial), eastern Tibetan Plateau, China; Middle Miocene 16–19 Ma	B(0.16, 0.19) — soft bounds following published formation age constraints	Zhang et al., 2019

B(min,max) denotes a soft uniform bound in MCMCtree; bounds here follow the ICS Lutetian window (47.8–41.2 Ma) and published formation ages for the Garang Fm. (16–19 Ma). ICS age window referenced for context. Kishenehn Eocene age is independently constrained, supporting the Lutetian minimum for Aphalaridae.

Table S5. Oligonucleotide primers used for SYBR Green qPCR and confirmatory conventional PCR (cPCR) targeting 16S rRNA genes of the primary symbiont "*Candidatus* Carsonella ruddii" (*Ca*Cr) and secondary symbionts in psyllids.

**Target (16S rRNA)**	**Symbiont status**	**Assay**	**Forward (5′–3′)**	**Reverse (5′–3′)**	**Amplicon (bp)**	**Host(s) screened**[d]
*Ca*Cr[a]	Primary	qPCR	TGACGACAGCCATGCAGC	CGGTGGAGCATGTGGTTTAA	129	All seven psyllid species
		cPCR	GAGACACGGCCCAAACTTTT	TGAGTTTTAACCTTGCGGCC	621	All seven psyllid species
"*Ca.* Profftella armatura"	Secondary	qPCR	CAAGTCGAACGGTAACAGAGAT	AATCAGATATCGGCCGCTCC	196	*Diaphorina citri*
		cPCR	ACACTGACGCTCATGTACGA	TGTATGACGTGTGAAGCCCT	487	*D. citri*
Wolbachia sp.	Secondary	qPCR	GGTAGTCCACGCTGTAAACG	CCCCAGGCGGAATGTTTAAC	87	*D. citri*, *Bactericera cockerelli*, *Cornegenapsylla sinica*
		cPCR	AACACATGCAAGTCGAACGG	CGGAGTTAGCCAGGACTTCT	427	*D. citri*, *Ba. cockerelli*, *Cor. sinica*
Enterobacteriaceae-like	Secondary	qPCR	TCCCCTACGGTTACCTTGTT	CTTGTACACACCGCCCGT	130	*Cacopsylla citrisuga*
		cPCR	AGCACTGTTTCGGGTAAGGA	ACAGCTTACAGAACGCTCCT	345	*Cac. citrisuga*
Unnamed S-endosymbiont 1[b]	Secondary	qPCR	TTTTGCAACCCACTCCCATG	CATGTCGCGGTGAATACGTT	70	*Macrohomotoma gladiata*
		cPCR	TCACATGAGCAAGCAGGGTA	CGTGTCCCACCCTACTCATT	526	*M. gladiata*
Unnamed S-endosymbiont 2[c]	Secondary	qPCR	CCCGTTACTTACATGCCAGC	CGTACCCCAAACCAACACAG	127	*Blastopsylla occidentalis*
		cPCR	ACGATGTGGGAAAGCTCAGA	CCCGTTACTTACATGCCAGC	742	*Bla. occidentalis*

[a] The Primary endosymbiont is "*Candidatus* Carsonella ruddii" for all listed psyllid species.

[b] Phylogenetically affiliated with the 16S rRNA gene of "*Candidatus* Arsenophonus nilaparvatae".

[c] Phylogenetically affiliated with the 16S rRNA gene of secondary endosymbiont of *Blastopsylla occidentalis* (GenBank accession no.: AF263558.1).

[d] No secondary endosymbiont was detected in *Cacopsylla chinensis* by either qPCR or cPCR using the assays listed above.

Table S6. Illumina sequencing yield and preliminary metagenomic *de novo* assembly statistics for the seven psyllid libraries.

**ID.**	**Psyllid species**	**Raw FASTQ size (GB)**	**Total raw reads**	**Preliminary *de novo* assembly: contigs (n)**	**Preliminary de novo assembly: N50 (bp)**
A	*Diaphorina citri*	29.5	8.36×10^{7}	62,650	1,624
B	*Bactericera cockerelli*	25.4	7.65×10^{7}	92,274	1,793
C	*Cacopsylla citrisuga*	26.3	7.55×10^{7}	61,570	1,541
D	*Cacopsylla chinensis*	29.8	8.85×10^{7}	56,208	1,470
E	*Cornegenapsylla sinica*	23.6	7.02×10^{7}	105,743	1,756
F	*Macrohomotoma gladiata*	24.9	7.4×10^{7}	68,455	1,646
G	*Blastopsylla occidentalis*	25.2	7.51×10^{7}	101,594	1,869

De novo assemblies were generated in CLC Genomics Workbench using the parameters described in the Methods (Section 2.2). These draft assemblies were used only for initial contig screening/read recruitment and are not intended as reference-quality host assemblies.

Table S7. Comparative BLAST analysis of "*Candidatus* Carsonella ruddii" genomes from seven psyllid species against GenBank.

Psyllid species	Query accession	Rank	Top BLAST hit description	Hit accession	Acc. len (bp)	Max score	Total score	Query cover (%)	E value	Per. identity (%)
Diaphorina citri	CP197248	1	*Candidatus* Carsonella ruddii isolate GDCZXQ chromosome, complete genome	CP146466.1	174021	3.206e+05	3.214e+05	100%	0.0	99.93%
	CP197248	2	*Candidatus* Carsonella ruddii isolate GDYN chromosome, complete genome	CP146454.1	174020	3.205e+05	3.213e+05	100%	0.0	99.91%
	CP197248	3	*Candidatus* Carsonella ruddii isolate GDMM chromosome, complete genome	CP146436.1	174021	3.205e+05	3.213e+05	100%	0.0	99.91%
Bactericera cockerelli	CP197249	1	MAG: *Candidatus* Carsonella ruddii isolate BC-CA chromosome, complete genome	CP167178.1	173994	3.213e+05	3.213e+05	100%	0.0	99.99%
	CP197249	2	*Candidatus* Carsonella ruddii strain BC, complete genome	CP019943.1	173802	1.688e+05	3.029e+05	100%	0.0	98.21%
	CP197249	3	*Candidatus* Carsonella ruddii strain BT chromosome	CP024798.1	174004	92337	2.235e+05	100%	0.0	90.51%
Cacopsylla citrisuga	CP197250	1	MAG: *Candidatus* Carsonella ruddii isolate CRpyc chromosome, complete genome	CP102600.1	168917	1.980e+05	2.379e+05	100%	0.0	92.08%
	CP197250	2	MAG: *Candidatus* Carsonella ruddii isolate CRmelAO3-2 chromosome, complete genome	CP102595.1	169081	1.864e+05	2.259e+05	100%	0.0	90.90%
	CP197250	3	MAG: *Candidatus* Carsonella ruddii isolate CRmelAO3-1 chromosome, complete genome	CP102596.1	169079	1.864e+05	2.259e+05	100%	0.0	90.90%
Cacopsylla chinensis	CP197251	1	MAG: *Candidatus* Carsonella ruddii isolate CRpyc chromosome, complete genome	CP102600.1	168917	2.552e+05	2.552e+05	100%	0.0	93.95%
	CP197251	2	MAG: *Candidatus* Carsonella ruddii isolate CRmelAO3-2 chromosome, complete genome	CP102595.1	169081	1.828e+05	2.209e+05	100%	0.0	90.44%
	CP197251	3	MAG: *Candidatus* Carsonella ruddii isolate CRmelAO3-1 chromosome, complete genome	CP102596.1	169079	1.828e+05	2.209e+05	100%	0.0	90.43%

Table S8. List of the 155 core genes shared by the seven newly assembled "*Candidatus* Carsonella ruddii" (*Ca*Cr) genomes, with predicted COG functional classifications.

NO.	CP197248	CP197249	CP197250	CP197251	CP197252	CP197253	CP197254	Total Copies	Gene Identifier (based on CP197248)	Functional Annotation	Predicted COG code
1	1	1	1	1	1	1	1	7	ACTJOE_00005	tRNA modification GTPase	A
2	1	1	1	1	1	1	1	7	ACTJOE_00010	FAD-dependent oxidoreductase	C
3	1	1	1	1	1	1	1	7	ACTJOE_00015	FOF1 ATP synthase subunit A	C
4	1	1	1	1	1	1	1	7	ACTJOE_00020	F0F1 ATP synthase subunit C	C
5	1	1	1	1	1	1	1	7	ACTJOE_00025	hypothetical protein	S
6	2	2	2	2	2	2	2	14	ACTJOE_00035	F0F1 ATP synthase subunit alpha	C
7	1	1	1	1	1	1	1	7	ACTJOE_00040	F0F1 ATP synthase subunit gamma	C
8	1	1	1	1	1	1	1	7	ACTJOE_00050	hypothetical protein	S
9	1	1	1	1	1	1	1	7	ACTJOE_00060	type II 3-dehydroquinate dehydratase	E
10	1	1	1	1	1	1	1	7	ACTJOE_00065	transketolase-like TK C-terminal-containing protein	G
11	1	1	1	1	2	1	1	8	ACTJOE_00070	hypothetical protein	S
12	1	1	1	1	1	1	1	7	ACTJOE_00075	methylenetetrahydrofolate reductase	H
13	1	1	1	1	1	1	1	7	ACTJOE_00095	DapH/DapD/GlmU-related protein	E
14	1	1	1	1	1	1	1	7	ACTJOE_00100	hypothetical protein	S
15	1	1	1	1	1	1	1	7	ACTJOE_00105	30S ribosomal protein S2	J
16	1	1	1	1	1	1	1	7	ACTJOE_00110	hypothetical protein	S
17	1	1	1	1	1	1	1	7	ACTJOE_00115	ribosome recycling factor	J
18	1	1	1	1	1	1	1	7	ACTJOE_00120	DNA polymerase III subunit alpha	L
19	1	1	1	1	1	1	1	7	ACTJOE_00125	hypothetical protein	S
20	1	1	1	1	1	1	1	7	ACTJOE_00135	phosphoribosyl-ATP pyrophosphatase	E
21	1	1	1	1	1	1	1	7	ACTJOE_00140	50S ribosomal protein L31	J
22	1	1	1	1	1	1	1	7	ACTJOE_00145	hypothetical protein	S
23	1	1	1	1	1	1	1	7	ACTJOE_00150	3-dehydroquinate synthase	E
24	1	1	1	1	1	1	1	7	ACTJOE_00155	dihydroxy-acid dehydratase	E
25	1	1	1	2	1	1	1	8	ACTJOE_00170	hypothetical protein	S
26	1	1	1	2	1	1	1	8	ACTJOE_00180	adenylosuccinate synthetase	F
27	1	1	1	1	1	1	1	7	ACTJOE_00185	Fe-S cluster assembly protein SufB	H
28	1	1	1	1	1	1	1	7	ACTJOE_00190	ATP-binding cassette domain-containing protein	O
29	2	2	2	3	2	1	2	14	ACTJOE_00195	hypothetical protein	S
30	1	1	1	1	1	1	1	7	ACTJOE_00200	aminotransferase class V-fold PLP-dependent enzyme	E
31	1	1	1	2	2	1	1	9	ACTJOE_00205	hypothetical protein	S
32	1	1	1	1	1	1	1	7	ACTJOE_00210	translation initiation factor IF-1	J
33	1	1	1	1	1	1	1	7	ACTJOE_00230	thioredoxin domain-containing protein	O
34	1	1	1	1	1	1	3	9	ACTJOE_00235	NAD(P)-binding domain-containing protein	R
35	1	1	1	1	1	1	1	7	ACTJOE_00270	hypothetical protein	S
36	1	1	1	1	1	1	1	7	ACTJOE_00275	ribose-phosphate diphosphokinase	E
37	2	2	2	2	2	2	2	14	ACTJOE_00280	peptide chain release factor-like protein	J
38	1	1	1	1	1	1	1	7	ACTJOE_00285	hypothetical protein	S
39	1	1	1	1	1	2	2	9	ACTJOE_00290	amidase family protein	O
40	2	1	2	2	1	1	1	10	ACTJOE_00300	hypothetical protein	S
41	1	1	1	1	1	1	1	7	ACTJOE_00305	histidinol dehydrogenase	E
42	1	1	1	1	1	1	1	7	ACTJOE_00310	uL13 family ribosomal protein	J
43	1	1	1	1	1	1	1	7	ACTJOE_00315	30S ribosomal protein S9	J
44	1	1	1	1	1	1	1	7	ACTJOE_00320	hypothetical protein	S
45	1	1	1	1	1	1	1	7	ACTJOE_00325	prephenate dehydratase domain-containing protein	E
46	1	1	1	1	1	1	1	7	ACTJOE_00330	3-phosphoshikimate 1-carboxyvinyltransferase	E
47	1	1	1	1	1	1	1	7	ACTJOE_00335	S1 RNA-binding domain-containing protein	J
48	1	1	1	1	1	1	1	7	ACTJOE_00340	chaperonin GroEL	O
49	1	1	1	1	1	1	1	7	ACTJOE_00345	co-chaperone GroES	O
50	1	1	1	1	1	1	1	7	ACTJOE_00360	TrmH family RNA methyltransferase	A
51	1	1	1	1	1	1	1	7	ACTJOE_00365	hypothetical protein	S
52	1	1	1	1	1	1	1	7	ACTJOE_00370	DnaB-like helicase C-terminal domain-containing protein	L
53	1	2	2	1	2	2	2	12	ACTJOE_00375	hypothetical protein	S
54	1	1	1	1	1	1	1	7	ACTJOE_00385	toprim domain-containing protein	L
55	1	1	1	1	1	1	1	7	ACTJOE_00390	sigma-70 family RNA polymerase sigma factor	K
56	1	1	1	1	1	1	1	7	ACTJOE_00400	aminotransferase class I/II-fold pyridoxal phosphate-dependent enzyme	E
57	2	2	2	2	2	2	2	14	ACTJOE_00405	imidazole glycerol phosphate synthase subunit HisF	E
58	1	1	1	1	1	1	1	7	ACTJOE_00415	imidazole glycerol phosphate synthase subunit HisH	E
59	1	1	1	1	1	1	1	7	ACTJOE_00420	hypothetical protein	S
60	1	1	1	1	1	1	1	7	ACTJOE_00440	hypothetical protein	S
61	1	1	1	1	1	1	1	7	ACTJOE_00445	nucleotide exchange factor GrpE	O
62	1	1	1	1	1	1	1	7	ACTJOE_00450	molecular chaperone DnaK	O
63	1	1	1	1	1	1	1	7	ACTJOE_00455	4-hydroxy-tetrahydrodipicolinate reductase	E
64	2	2	2	2	2	1	2	13	ACTJOE_00480	GTP-binding protein	T
65	1	1	1	1	1	1	1	7	ACTJOE_00490	hypothetical protein	S
66	2	3	2	2	2	2	2	15	ACTJOE_00495	glutamate--tRNA ligase family protein	J
67	1	1	1	1	1	1	1	7	ACTJOE_00515	ATP-dependent Clp protease proteolytic subunit	O
68	1	1	1	1	1	1	1	7	ACTJOE_00520	ATP-dependent Clp protease ATP-binding subunit	O
69	1	1	1	2	1	1	2	9	ACTJOE_00525	phospholipase D-like domain-containing protein	I
70	1	1	1	2	1	1	1	8	ACTJOE_00530	amino acid--tRNA ligase-related protein	J
71	1	1	1	1	2	1	1	8	ACTJOE_00545	hypothetical protein	S
72	1	1	1	1	1	1	1	7	ACTJOE_00550	bifunctional proline dehydrogenase/L	C
73	1	1	1	1	1	1	1	7	ACTJOE_00555	hypothetical protein	S
74	1	1	1	1	1	1	1	7	ACTJOE_00560	chorismate synthase	E
75	1	1	1	1	1	1	1	7	ACTJOE_00565	aconitase family protein	E
76	1	1	1	1	1	1	1	7	ACTJOE_00570	3-isopropylmalate dehydratase small subunit	E
77	1	1	1	1	1	1	1	7	ACTJOE_00575	3-isopropylmalate dehydrogenase	E
78	1	1	1	1	1	1	1	7	ACTJOE_00580	aspartate-semialdehyde dehydrogenase	E
79	1	1	1	1	1	1	1	7	ACTJOE_00590	aminoacyl--tRNA ligase-related protein	J
80	1	1	1	1	1	1	1	7	ACTJOE_00600	SufE family protein	P

Table S8 (continued)

81	1	1	1	1	1	1	1	7	ACTJOE_00605	phosphoribosyl-AMP cyclohydrolase	E
82	1	1	1	1	1	1	1	7	ACTJOE_00615	exonuclease domain-containing protein	A
83	1	1	1	2	1	1	1	8	ACTJOE_00625	tRNA ligase subunit PheS family protein	J
84	1	1	1	1	1	1	1	7	ACTJOE_00630	50S ribosomal protein L20	J
85	1	1	1	1	1	1	1	7	ACTJOE_00640	hypothetical protein	S
86	1	1	1	1	1	1	1	7	ACTJOE_00645	superoxide dismutase	O
87	1	1	1	1	1	1	1	7	ACTJOE_00660	3-deoxy-7-phosphoheptulonate synthase	E
88	1	1	1	1	1	1	1	7	ACTJOE_00670	MiaB/RimO family radical SAM methylthiotransferase	A
89	1	1	1	1	1	2	1	8	ACTJOE_00675	hypothetical protein	S
90	4	4	4	4	4	6	4	30	ACTJOE_00680	class I tRNA ligase family protein	J
91	1	1	1	1	1	1	1	7	ACTJOE_00685	succinate--CoA ligase subunit alpha	C
92	1	1	1	2	1	1	1	8	ACTJOE_00690	ATP-grasp domain-containing protein	E
93	1	1	1	1	1	1	1	7	ACTJOE_00720	argininosuccinate synthase	E
94	1	1	1	1	1	1	1	7	ACTJOE_00735	dihydrodipicolinate synthase family protein	E
95	1	1	1	1	1	1	1	7	ACTJOE_00750	malate:quinone oxidoreductase	C
96	1	1	1	1	1	1	1	7	ACTJOE_00765	aspartate kinase	E
97	1	1	1	1	1	1	1	7	ACTJOE_00775	recombinase RecA	L
98	1	1	1	1	1	1	1	7	ACTJOE_00785	redoxin domain-containing protein	O
99	1	1	1	1	1	1	1	7	ACTJOE_00790	transaldolase family protein	G
100	1	1	1	1	1	1	1	7	ACTJOE_00820	hypothetical protein	S
101	1	1	1	1	1	1	1	7	ACTJOE_00825	50S ribosomal protein L33	J
102	1	1	1	1	1	1	1	7	ACTJOE_00830	L28 family ribosomal protein	J
103	1	1	1	1	1	1	1	7	ACTJOE_00835	hypothetical protein	S
104	1	1	1	1	1	1	1	7	ACTJOE_00850	threonine synthase	E
105	1	1	1	1	1	1	1	7	ACTJOE_00855	hypothetical protein	S
106	1	1	1	1	1	1	1	7	ACTJOE_00860	hypothetical protein	S
107	1	1	1	1	1	1	1	7	ACTJOE_00870	30S ribosomal protein S16	J
108	1	1	1	1	1	1	1	7	ACTJOE_00880	ketol-acid reductoisomerase	E
109	1	1	1	1	2	1	1	8	ACTJOE_00885	hypothetical protein	S
110	1	1	1	1	1	1	1	7	ACTJOE_00890	thiamine pyrophosphate-dependent enzyme	H
111	3	1	1	1	1	1	1	9	ACTJOE_00895	pseudouridine synthase	A
112	1	1	1	1	1	1	1	7	ACTJOE_00915	hypothetical protein	S
113	1	1	1	1	1	1	1	7	ACTJOE_00920	hypothetical protein	S
114	1	1	1	1	1	1	1	7	ACTJOE_00925	50S ribosomal protein L27	J
115	1	1	1	1	1	1	1	7	ACTJOE_00930	bL21 family ribosomal protein	J
116	1	1	1	1	1	1	1	7	ACTJOE_00935	hypothetical protein	S
117	1	1	1	1	1	1	1	7	ACTJOE_00940	DNA-directed RNA polymerase subunit alpha C	K
118	1	1	1	1	1	1	1	7	ACTJOE_00945	30S ribosomal protein S4	J
119	1	1	1	1	1	1	1	7	ACTJOE_00950	30S ribosomal protein S11	J
120	1	1	1	1	1	1	1	7	ACTJOE_00955	ribosomal protein uS13	J
121	1	1	1	1	1	1	1	7	ACTJOE_00960	50S ribosomal protein L36	J
122	1	1	1	1	1	1	1	7	ACTJOE_00965	50S ribosomal protein L15	J
123	1	1	1	1	1	1	1	7	ACTJOE_00970	hypothetical protein	S
124	1	1	1	1	1	1	1	7	ACTJOE_00980	hypothetical protein	S
125	1	1	1	1	1	1	1	7	ACTJOE_00985	30S ribosomal protein S8	J
126	1	1	1	1	1	1	1	7	ACTJOE_00990	hypothetical protein	S
127	1	1	1	1	1	1	1	7	ACTJOE_00995	50S ribosomal protein L5	J
128	1	1	1	1	1	1	1	7	ACTJOE_01000	50S ribosomal protein L14	J
129	1	1	1	1	1	1	1	7	ACTJOE_01005	small ribosomal subunit protein uS17	J
130	1	1	1	1	1	1	1	7	ACTJOE_01010	50S ribosomal protein L16	J
131	1	1	1	1	1	1	1	7	ACTJOE_01015	30S ribosomal protein S3	J
132	1	1	1	1	1	1	1	7	ACTJOE_01020	uL22 family ribosomal protein	J
133	1	1	1	1	1	1	1	7	ACTJOE_01025	30S ribosomal protein S19	J
134	1	1	1	1	1	1	1	7	ACTJOE_01030	50S ribosomal protein L2	J
135	1	1	1	1	1	1	1	7	ACTJOE_01035	50S ribosomal protein L4	J
136	1	1	1	1	1	1	1	7	ACTJOE_01040	50S ribosomal protein L3	J
137	1	1	1	1	1	1	1	7	ACTJOE_01045	30S ribosomal protein S10	J
138	2	2	2	2	2	2	2	14	ACTJOE_01050	elongation factor Tu	J
139	1	1	1	1	1	1	1	7	ACTJOE_01060	30S ribosomal protein S7	J
140	1	1	1	1	1	1	1	7	ACTJOE_01065	30S ribosomal protein S12	J
141	1	1	1	1	1	1	1	7	ACTJOE_01070	DNA-directed RNA polymerase subunit beta	K
142	1	1	1	1	1	1	1	7	ACTJOE_01075	DNA-directed RNA polymerase subunit beta'	K
143	1	1	1	1	1	1	1	7	ACTJOE_01080	ribosomal protein bL12	J
144	1	1	1	1	1	1	1	7	ACTJOE_01085	hypothetical protein	S
145	1	1	1	1	1	1	2	8	ACTJOE_01090	hypothetical protein	S
146	1	1	1	1	1	1	1	7	ACTJOE_01095	uL11 family ribosomal protein	J
147	1	1	1	1	1	1	1	7	ACTJOE_01120	hypothetical protein	S
148	1	1	1	1	1	1	1	7	ACTJOE_01125	cbb3-type cytochrome c oxidase subunit I	C
149	1	1	1	1	1	1	1	7	ACTJOE_01130	hypothetical protein	S
150	1	1	1	1	1	1	1	7	ACTJOE_01140	aminotransferase class IV	E
151	1	1	1	1	1	1	1	7	ACTJOE_01150	peptide deformylase	O
152	1	1	1	1	1	1	1	7	ACTJOE_01160	diaminopimelate decarboxylase family protein	E
153	1	1	1	1	1	1	1	7	ACTJOE_01165	hypothetical protein	S
154	1	1	1	1	1	1	1	7	ACTJOE_01170	glycine--tRNA ligase subunit alpha	J
155	1	1	1	1	1	1	1	7	ACTJOE_01180	cold-shock protein	A

Abbreviations: COG, Clusters of Orthologous Groups. Functional category assignments were standardized to ensure agreement with the total counts and proportions shown in Fig. 3A. COG functional categories: [A] RNA processing and modification; [C] energy production and conversion; [E] amino acid transport and metabolism; [F] nucleotide transport and metabolism; [G] carbohydrate transport and metabolism; [H] coenzyme transport and metabolism; [I] lipid transport and metabolism; [J] translation, ribosomal structure and biogenesis; [K] transcription; [L] replication, recombination and repair; [O] post-translational modification, protein turnover, chaperones; [P] inorganic ion transport and metabolism; [R] general function prediction only; [S] function unknown; [T] signal transduction mechanisms.

Table S9. Secondary symbionts detected in psyllids and BLASTn identification metrics (16S/IGS/23S).

ID	Psyllid host	Secondary symbiont (locus)	This study: sequence ID	Best BLAST hit (species)	Best-hit accession	Max score	Query coverage (%)	Percent identity (%)	E-value
A	*Diaphorina citri*	*Wolbachia* symbiont (16S rRNA)	PX124078	*Wolbachia* symbiont	OQ102148.1	2765	100	99.91	0.0
		"*Candidatus* Profftella armatura" (16S–IGS–23S)	PX118601	"*Ca.* Profftella armatura"	CP146408.1	8455	100	100	0.0
B	*Bactericera cockerelli*	*Wolbachia* symbiont (16S rRNA)	PX124079	*Wolbachia* symbiont	PP808502.1	2771	100	100	0.0
C	*Cacopsylla citrisuga*	Uncultured secondary symbiont (16S–IGS–23S), *Arsenophonus*-like	PX118602	Enterobacteriaceae bacterium (Arsenophonus-like)	CP116499.1	7322	100	94.12	0
D	*Cacopsylla chinensis*	Not detected	—	—	—	—	—	—	—
E	*Cornegenapsylla sinica*	*Wolbachia* symbiont (16S rRNA)	PX124080	*Wolbachia* symbiont	OQ102146.1	2669	100	99.46	0.0
F	*Macrohomotoma gladiata*	Secondary symbiont (16S–IGS–23S), *Arsenophonus*-like	PX113536	"*Ca.* Arsenophonus nilaparvatae"	CP158507.1	7810	100	95.04	0
G	*Blastopsylla occidentalis*	Secondary symbiont (16S–IGS–23S)	PX113323	Secondary symbiont of *Blastopsylla occidentalis*	AF263558.1	8585	100	99.89	0

Abbreviations: IGS, intergenic spacer.

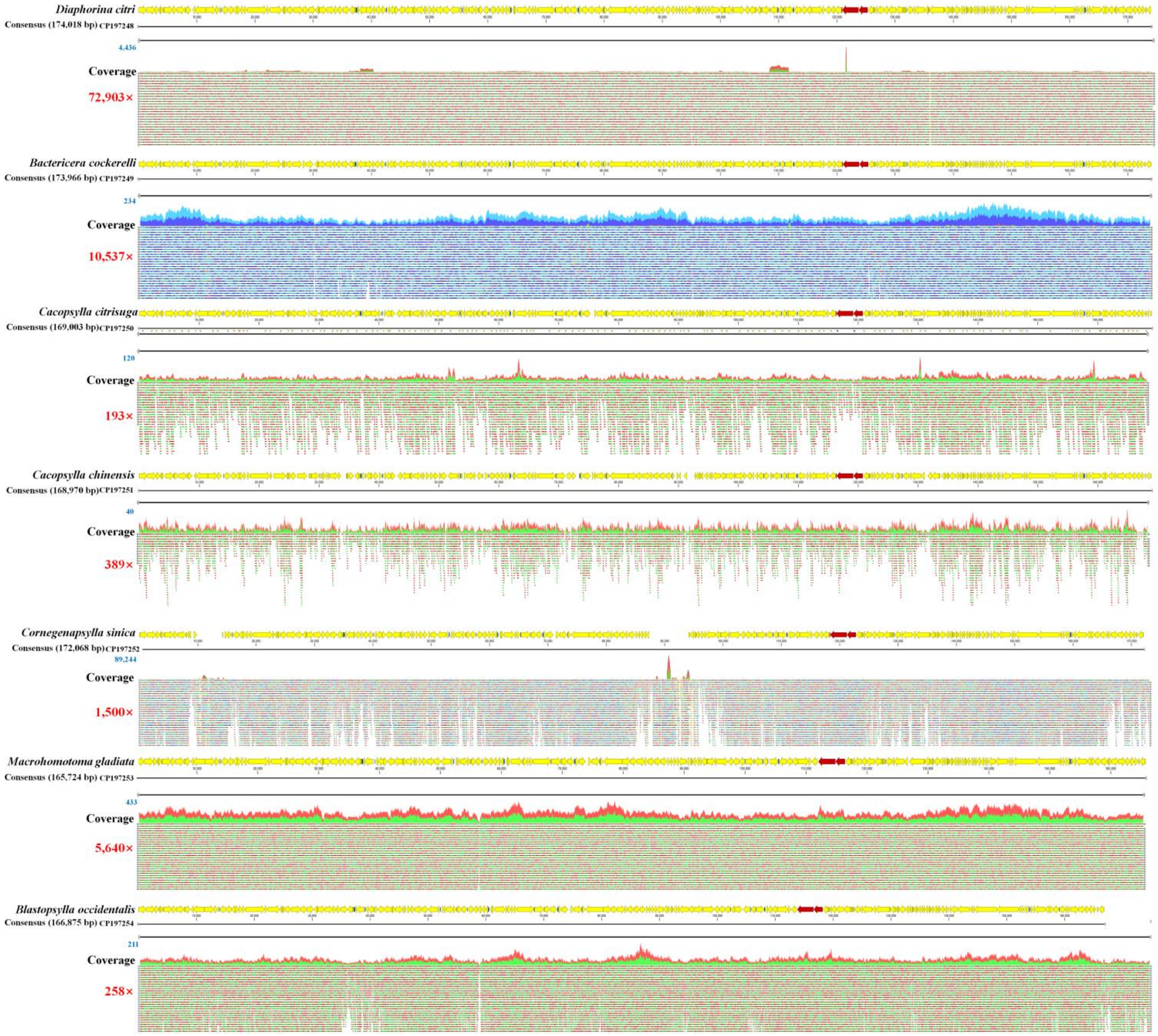

Fig. S1. Strand-specific sequencing coverage and consensus tracks of '*Candidatus* Carsonella ruddii' (*Ca*Cr) genomes across seven psyllid species. Illumina HiSeq 150-bp reads were mapped to the *Candidatus Carsonella ruddii* assemblies from the following seven psyllid species: *Diaphorina citri* (CP197248), *Bactericera cockerelli* (CP197249), *Cacopsylla citrisuga* (CP197250), *Cacopsylla chinensis* (CP197251), *Cornegenapsylla sinica* (CP197252), *Macrohomotoma gladiata* (CP197253), and *Blastopsylla occidentalis* (CP197254). Functional annotation was performed using the NCBI Prokaryotic Genome Annotation Pipeline (PGAP). Yellow represents protein-coding genes, red represents rRNA genes, and blue represents tRNA genes. Coverage plots show maximum depth values (indicated in blue text on the top scale) and average coverage (indicated in red text). Forward and reverse reads are distinguished by different colors.

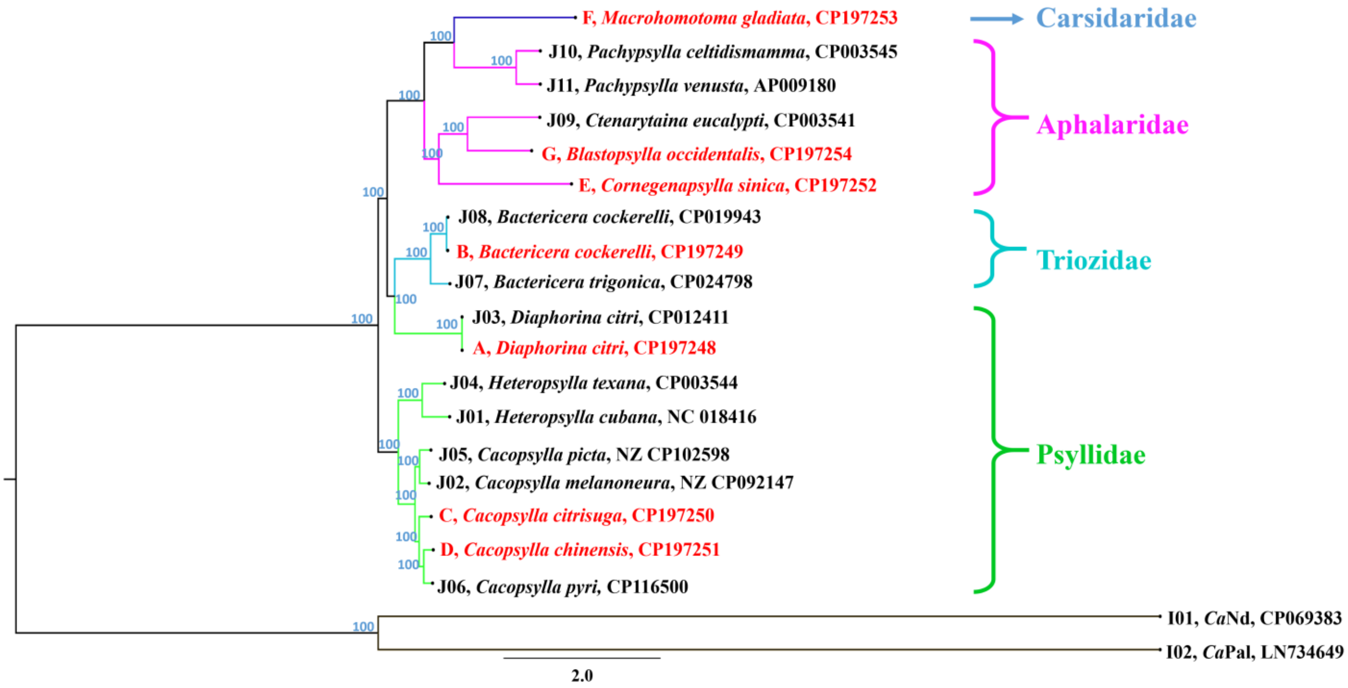

Fig. S2. Phylogeny of "*Candidatus* Carsonella ruddii" primary symbionts across psyllid species using maximum likelihood (ML). Branches are colour-coded by family (labels at right), and taxa newly sequenced in this study are highlighted in red. Node values show bootstrap values. Outgroups: "*Ca*. Nardonella dryophthoridicola" (*Ca*Nd) from the red palm weevil *Rhynchophorus ferrugineus*, and "*Ca*. Portiera aleyrodidarum" (*Ca*Pal) from the whitefly *Aleurodicus floccissimus*.

Supplementary Code S1. Python script for calculating intergenic-region statistics from GenBank files

```
#!/usr/bin/env python3
"""
Supplementary code: calculate intergenic-region statistics from GenBank files.

What this script reports for each GenBank record:
1. Number of Intergenic Regions
2. Intergenic Region Length Range (bp)
3. Additional useful summary columns for transparency:
    - Total Intergenic Length (bp)
    - Mean Intergenic Length (bp)
    - Genome Length (bp)
    - Number of Gene Features Used
Behavior:
- If no input files are given on the command line, the script automatically scans
  the same folder as this script for .gbk, .gbff, .gb, and .genbank files.
- Output is English-only and suitable for supplementary materials.
Recommended interpretation:
- Intergenic regions are computed from merged gene intervals.
- By default, the script uses "gene" features.
- If a record has no "gene" features, it falls back to CDS/tRNA/rRNA/ncRNA/tmRNA/
  misc_RNA/pseudogene.
"""
from __future__ import annotations

import argparse
import csv
import sys
from dataclasses import dataclass
from pathlib import Path
from statistics import mean
from typing import Iterable, List, Sequence, Tuple
from Bio import SeqIO
from Bio.SeqFeature import CompoundLocation, SimpleLocation
FALLBACK_FEATURE_TYPES = (
    "CDS",
    "tRNA",
    "rRNA",
    "ncRNA",
    "tmRNA",
    "misc_RNA",
    "pseudogene",
)
```

```
@dataclass
class GenomicInterval:
    start: int    # 0-based inclusive
    end: int        # 0-based exclusive
    @property
    def length(self) -> int:
        return self.end - self.start
@dataclass
class IntergenicRegion:
    record_id: str
    region_index: int
    start_1based: int
    end_1based: int
    length_bp: int
    wraps_origin: bool
@dataclass
class RecordSummary:
    record_id: str
    genome_length_bp: int
    topology: str
    feature_source: str
    feature_count_used: int
    number_of_intergenic_regions: int
    min_intergenic_length_bp: int
    max_intergenic_length_bp: int
    intergenic_region_length_range_bp: str
    total_intergenic_length_bp: int
    mean_intergenic_length_bp: float
def parse_args() -> argparse.Namespace:
    parser = argparse.ArgumentParser(
        description="Calculate the number of intergenic regions and intergenic region length range (bp) from GenBank
files."
    )
    parser.add_argument(
        "genbank",
        nargs="*",
        help="Optional input GenBank files. If omitted, files in the script folder will be scanned automatically.",
    )
    parser.add_argument(
        "--output-prefix",
        default="intergenic_region_statistics",
        help="Prefix for output TSV files.",
    )
    parser.add_argument(
        "--feature-preference",
        choices=["gene", "auto"],
        default="auto",
        help=(
```

```
                'Feature selection strategy. "auto" uses gene features when available, '
                'otherwise falls back to CDS/tRNA/rRNA/ncRNA/tmRNA/misc_RNA/pseudogene. '
                '"gene" forces use of gene features only.'
            ),
    )
    return parser.parse_args()
def auto_find_genbank_files() -> List[Path]:
    script_dir = Path(__file__).resolve().parent
    patterns = ("*.gbk", "*.gbff", "*.gb", "*.genbank")
    files: List[Path] = []
    for pattern in patterns:
        files.extend(script_dir.glob(pattern))
    return sorted(set(path.resolve() for path in files))
def location_bounds(location: SimpleLocation | CompoundLocation) -> Tuple[int, int]:
    if isinstance(location, CompoundLocation):
        starts = [int(part.start) for part in location.parts]
        ends = [int(part.end) for part in location.parts]
        return min(starts), max(ends)
    return int(location.start), int(location.end)
def merge_intervals(intervals: Iterable[Tuple[int, int]]) -> List[Tuple[int, int]]:
    sorted_intervals = sorted(intervals)
    if not sorted_intervals:
        return []
    merged: List[List[int]] = [[sorted_intervals[0][0], sorted_intervals[0][1]]]
    for start, end in sorted_intervals[1:]:
        last_start, last_end = merged[-1]
        if start <= last_end:
            merged[-1][1] = max(last_end, end)
        else:
            merged.append([start, end])
    return [(start, end) for start, end in merged]
def choose_feature_intervals(record, feature_preference: str) -> Tuple[List[GenomicInterval], str]:
    gene_intervals: List[GenomicInterval] = []
    fallback_intervals: List[GenomicInterval] = []
    for feature in record.features:
        try:
            start, end = location_bounds(feature.location)
        except Exception:
            continue
        if end <= start:
            continue
        if feature.type == "gene":
            gene_intervals.append(GenomicInterval(start, end))
        elif feature.type in FALLBACK_FEATURE_TYPES:
            fallback_intervals.append(GenomicInterval(start, end))
    if feature_preference == "gene":
        return gene_intervals, "gene"
    if gene_intervals:
```

```
        return gene_intervals, "gene"
    return fallback_intervals, "fallback_annotation_features"
def compute_intergenic_regions(
    record_id: str,
    genome_length: int,
    merged_intervals: Sequence[Tuple[int, int]],
    circular: bool,
) -> List[IntergenicRegion]:
    if not merged_intervals:
        return []
    regions: List[IntergenicRegion] = []
    if circular:
        for i in range(len(merged_intervals)):
            current_start, current_end = merged_intervals[i]
            next_start, next_end = merged_intervals[(i + 1) % len(merged_intervals)]
            if i == len(merged_intervals) - 1:
                gap = (genome_length - current_end) + next_start
                if gap > 0:
                    start_1based = current_end + 1
                    end_1based = next_start if next_start > 0 else genome_length
                    regions.append(
                        IntergenicRegion(
                            record_id=record_id,
                            region_index=len(regions) + 1,
                            start_1based=start_1based,
                            end_1based=end_1based,
                            length_bp=gap,
                            wraps_origin=True,
                        )
                    )
            else:
                gap = next_start - current_end
                if gap > 0:
                    regions.append(
                        IntergenicRegion(
                            record_id=record_id,
                            region_index=len(regions) + 1,
                            start_1based=current_end + 1,
                            end_1based=next_start,
                            length_bp=gap,
                            wraps_origin=False,
                        )
                    )
    else:
        # Gap before the first feature
        first_start, first_end = merged_intervals[0]
        if first_start > 0:
            regions.append(
```

```
                    IntergenicRegion(
                        record_id=record_id,
                        region_index=len(regions) + 1,
                        start_1based=1,
                        end_1based=first_start,
                        length_bp=first_start,
                        wraps_origin=False,
                    )
                )
        # Gaps between adjacent features
        for i in range(len(merged_intervals) - 1):
            current_start, current_end = merged_intervals[i]
            next_start, next_end = merged_intervals[i + 1]
            gap = next_start - current_end
            if gap > 0:
                regions.append(
                    IntergenicRegion(
                        record_id=record_id,
                        region_index=len(regions) + 1,
                        start_1based=current_end + 1,
                        end_1based=next_start,
                        length_bp=gap,
                        wraps_origin=False,
                    )
                )
        # Gap after the last feature
        last_start, last_end = merged_intervals[-1]
        terminal_gap = genome_length - last_end
        if terminal_gap > 0:
            regions.append(
                IntergenicRegion(
                    record_id=record_id,
                    region_index=len(regions) + 1,
                    start_1based=last_end + 1,
                    end_1based=genome_length,
                    length_bp=terminal_gap,
                    wraps_origin=False,
                )
            )
    return regions
def summarize_record(
    record,
    feature_source: str,
    intervals: Sequence[GenomicInterval],
    intergenic_regions: Sequence[IntergenicRegion],
) -> RecordSummary:
    lengths = [region.length_bp for region in intergenic_regions]
    min_len = min(lengths) if lengths else 0
```

```
    max_len = max(lengths) if lengths else 0
    range_text = f"{min_len}-{max_len}" if lengths else "0-0"
    return RecordSummary(
        record_id=record.id,
        genome_length_bp=len(record.seq),
        topology=str(record.annotations.get("topology", "unknown")).lower(),
        feature_source=feature_source,
        feature_count_used=len(intervals),
        number_of_intergenic_regions=len(intergenic_regions),
        min_intergenic_length_bp=min_len,
        max_intergenic_length_bp=max_len,
        intergenic_region_length_range_bp=range_text,
        total_intergenic_length_bp=sum(lengths),
        mean_intergenic_length_bp=mean(lengths) if lengths else 0.0,
    )
def write_detail_output(path: Path, rows: Sequence[IntergenicRegion]) -> None:
    with path.open("w", newline="", encoding="utf-8") as handle:
        writer = csv.writer(handle, delimiter="\t")
        writer.writerow(
            [
                "record_id",
                "region_index",
                "start_1based",
                "end_1based",
                "length_bp",
                "wraps_origin",
            ]
        )
        for row in rows:
            writer.writerow(
                [
                    row.record_id,
                    row.region_index,
                    row.start_1based,
                    row.end_1based,
                    row.length_bp,
                    str(row.wraps_origin),
                ]
            )
def write_summary_output(path: Path, rows: Sequence[RecordSummary]) -> None:
    with path.open("w", newline="", encoding="utf-8") as handle:
        writer = csv.writer(handle, delimiter="\t")
        writer.writerow(
            [
                "record_id",
                "genome_length_bp",
                "topology",
                "feature_source",
```

```
                "feature_count_used",
                "number_of_intergenic_regions",
                "min_intergenic_length_bp",
                "max_intergenic_length_bp",
                "intergenic_region_length_range_bp",
                "total_intergenic_length_bp",
                "mean_intergenic_length_bp",
            ]
        )
        for row in rows:
            writer.writerow(
                [
                    row.record_id,
                    row.genome_length_bp,
                    row.topology,
                    row.feature_source,
                    row.feature_count_used,
                    row.number_of_intergenic_regions,
                    row.min_intergenic_length_bp,
                    row.max_intergenic_length_bp,
                    row.intergenic_region_length_range_bp,
                    row.total_intergenic_length_bp,
                    f"{row.mean_intergenic_length_bp:.4f}",
                ]
            )
def main() -> None:
    args = parse_args()
    if args.genbank:
        input_files = [Path(item) for item in args.genbank]
    else:
        input_files = auto_find_genbank_files()
    if not input_files:
        print("Error: no GenBank files were found.")
        print("Place .gbk/.gbff/.gb/.genbank files in the same folder as this script, or provide file paths explicitly.")
        sys.exit(1)
    all_regions: List[IntergenicRegion] = []
    all_summaries: List[RecordSummary] = []
    print("Input files:")
    for path in input_files:
        print(f" - {path}")
    for gbk_file in input_files:
        try:
            records = list(SeqIO.parse(str(gbk_file), "genbank"))
        except Exception as exc:
            print(f"Failed to read file: {gbk_file}")
            print(f"Reason: {exc}")
            continue
        if not records:
```

```
                print(f"No GenBank records found in: {gbk_file}")
                continue
            for record in records:
                intervals, feature_source = choose_feature_intervals(record, args.feature_preference)
                if not intervals:
                    summary = RecordSummary(
                        record_id=record.id,
                        genome_length_bp=len(record.seq),
                        topology=str(record.annotations.get("topology", "unknown")).lower(),
                        feature_source=feature_source,
                        feature_count_used=0,
                        number_of_intergenic_regions=0,
                        min_intergenic_length_bp=0,
                        max_intergenic_length_bp=0,
                        intergenic_region_length_range_bp="0-0",
                        total_intergenic_length_bp=0,
                        mean_intergenic_length_bp=0.0,
                    )
                    all_summaries.append(summary)
                    continue
                merged = merge_intervals((item.start, item.end) for item in intervals)
                topology = str(record.annotations.get("topology", "unknown")).lower()
                circular = topology == "circular"
                regions = compute_intergenic_regions(
                    record_id=record.id,
                    genome_length=len(record.seq),
                    merged_intervals=merged,
                    circular=circular,
                )
                summary = summarize_record(
                    record=record,
                    feature_source=feature_source,
                    intervals=intervals,
                    intergenic_regions=regions,
                )
                all_regions.extend(regions)
                all_summaries.append(summary)
    output_dir = Path(__file__).resolve().parent
    detail_path = output_dir / f"{args.output_prefix}_details.tsv"
    summary_path = output_dir / f"{args.output_prefix}_summary.tsv"
    write_detail_output(detail_path, all_regions)
    write_summary_output(summary_path, all_summaries)
    print("\nFinished.")
    print(f"Summary output: {summary_path}")
    print(f"Detail output:    {detail_path}")
if __name__ == "__main__":
    main()
```

Supplementary Code S2. R script for accessory-gene heatmap visualization

```
# Binary accessory-gene presence/absence heatmap for manuscript figures
#
# Purpose:
#     Read a binary presence/absence matrix from an Excel worksheet and
#     generate a publication-ready clustered heatmap using pheatmap.
#
# Input requirements:
#     1. An Excel workbook (.xlsx)
#     2. The first column contains gene identifiers (default column name: Gene)
#     3. Remaining columns contain binary values (0 = absent, 1 = present)
#
# Output files:
#     1. A vector PDF for manuscript editing and production
#     2. A high-resolution TIFF for journal submission
#
# Example:
#     input_file    <- "Orthogroup.xlsx"
#     sheet_name    <- "Sheet3"
#     gene_column <- "Gene"
#
# Required packages:
#     install.packages(c("readxl", "pheatmap"))

suppressPackageStartupMessages({
  library(readxl)
  library(pheatmap)
})

# ============================
# User-defined parameters
# ============================
input_file    <- "Orthogroup.xlsx"
sheet_name    <- "Sheet3"
gene_column <- "Gene"
output_prefix <- "Figure_4_accessory_gene_presence_absence_heatmap"
# Heatmap appearance
absence_color <- "#BFBFBF"      # gray for absence (0)
presence_color <- "#FF9900"    # orange for presence (1)
cluster_rows <- TRUE
cluster_cols <- TRUE
show_rownames <- TRUE
show_colnames <- TRUE
border_color <- "black"
fontsize_row <- 8
fontsize_col <- 10
cellwidth <- 20
```

```r
cellheight <- 7
angle_col <- 45
# Output size
pdf_width    <- 6
pdf_height <- 8
tiff_width <- 6
tiff_height <- 8
tiff_res <- 600
# ============================
# Read and validate input data
# ============================
raw_data <- as.data.frame(read_xlsx(path = input_file, sheet = sheet_name))

if (!(gene_column %in% colnames(raw_data))) {
   stop(sprintf("Column '%s' was not found in the worksheet.", gene_column))
}
rownames(raw_data) <- raw_data[[gene_column]]
heatmap_data <- raw_data[, setdiff(colnames(raw_data), gene_column), drop = FALSE]

# Convert all remaining columns to numeric
heatmap_data[] <- lapply(heatmap_data, function(x) as.numeric(as.character(x)))
heatmap_matrix <- as.matrix(heatmap_data)
storage.mode(heatmap_matrix) <- "numeric"

# Check whether the matrix is binary
valid_values <- unique(as.vector(heatmap_matrix))
valid_values <- valid_values[!is.na(valid_values)]
if (!all(valid_values %in% c(0, 1))) {
   warning("The matrix contains values other than 0 and 1. Please verify the input data.")
}
# ============================
# Common plotting arguments
# ============================
heatmap_args <- list(
   mat = heatmap_matrix,
   color = c(absence_color, presence_color),
   breaks = c(-0.5, 0.5, 1.5),
   cluster_rows = cluster_rows,
   cluster_cols = cluster_cols,
   border_color = border_color,
   legend = TRUE,
   legend_breaks = c(0, 1),
   legend_labels = c("Absent", "Present"),
   show_rownames = show_rownames,
   show_colnames = show_colnames,
   fontsize_row = fontsize_row,
   fontsize_col = fontsize_col,
   cellwidth = cellwidth,
```

```
    cellheight = cellheight,
    angle_col = angle_col,
    na_col = "white"
)
# ==============================
# Export vector PDF
# ==============================
pdf_file <- paste0(output_prefix, ".pdf")
pdf(pdf_file, width = pdf_width, height = pdf_height, useDingbats = FALSE)
do.call(pheatmap, heatmap_args)
dev.off()
# ==============================
# Export high-resolution TIFF
# ==============================
tiff_file <- paste0(output_prefix, ".tiff")
tiff(
    filename = tiff_file,
    width = tiff_width,
    height = tiff_height,
    units = "in",
    res = tiff_res,
    compression = "lzw"
)
do.call(pheatmap, heatmap_args)
dev.off()
message("Heatmap files saved as:")
message("    ", pdf_file)
message("    ", tiff_file)
```

Supplementary References

Ouvrard D, Burckhardt D, Greenwalt D. The oldest jumping plant-louse (Insecta: Hemiptera: Sternorrhyncha) with comments on the classification and nomenclature of the Palaeogene Psylloidea. Acta Musei Moraviae, Scientiae Biologicae. 2013;98(2):21-33.

Petrone JR, Muñoz-Beristain A, Glusberger PR, Russell JT, Triplett EW. Unamplified, long-read metagenomic sequencing approach to close endosymbiont genomes of low-biomass insect populations. Microorganisms. 2022;10(3):513. doi:10.3390/microorganisms10030513.

Wu F, Deng X, Liang G, Huang J, Cen Y, Chen J. Whole-genome sequence of "*Candidatus* Profftella armatura" from *Diaphorina citri* in Guangdong, China. Genome Announc. 2015;3(6):e01282-15. doi:10.1128/genomeA.01282-15.
